# iTRAQ-based proteome profiling revealed the role of Phytochrome A in regulating primary metabolism in tomato seedling

**DOI:** 10.1038/s41598-021-87208-9

**Published:** 2021-04-06

**Authors:** Sherinmol Thomas, Rakesh Kumar, Kapil Sharma, Abhilash Barpanda, Yellamaraju Sreelakshmi, Rameshwar Sharma, Sanjeeva Srivastava

**Affiliations:** 1 Proteomics Lab, Department of Biosciences and Bioengineering, IIT Bombay, Mumbai, Maharashtra 400076 India; 2 Repository of Tomato Genomics Resources, Department of Plant Sciences, University of Hyderabad, Hyderabad, 500046 India; 3 Deptartment of Life Science, Central University of Karnataka, Kadaganchi, Kalaburagi, Karnataka 585367 India

**Keywords:** Mass spectrometry, Proteomic analysis, Plant sciences

## Abstract

In plants, during growth and development, photoreceptors monitor fluctuations in their environment and adjust their metabolism as a strategy of surveillance. Phytochromes (Phys) play an essential role in plant growth and development, from germination to fruit development. FR-light (FR) insensitive mutant (*fri*) carries a recessive mutation in Phytochrome A and is characterized by the failure to de-etiolate in continuous FR. Here we used iTRAQ-based quantitative proteomics along with metabolomics to unravel the role of Phytochrome A in regulating central metabolism in tomato seedlings grown under FR. Our results indicate that Phytochrome A has a predominant role in FR-mediated establishment of the mature seedling proteome. Further, we observed temporal regulation in the expression of several of the late response proteins associated with central metabolism. The proteomics investigations identified a decreased abundance of enzymes involved in photosynthesis and carbon fixation in the mutant. Profound accumulation of storage proteins in the mutant ascertained the possible conversion of sugars into storage material instead of being used or the retention of an earlier profile associated with the mature embryo. The enhanced accumulation of organic sugars in the seedlings indicates the absence of photomorphogenesis in the mutant.

## Introduction

Plant development is intimately bound to the external light environment. Light drives photosynthetic carbon fixation and activates a set of signal-transducing photoreceptors that regulate plant growth and development. Plants have evolved an extraordinary degree of developmental plasticity to optimize their growth and metabolism in response to the changing environmental conditions. To sense different features of light such as quality, quantity, direction, duration of photoperiod, and integration of these signals to initiate the appropriate physiological and developmental response; plants have evolved a diverse set of photoreceptors^1,2^. Phytochromes that sense red (R) and far-red light (FR)^3^ are photoreceptors with which plants gather environmental information and play a critical role in growth and development. Phytochrome A (PhyA), a photolabile phytochrome, is a unique photoreceptor which mediates FR high irradiance response (FR-HIR) of etiolated seedlings^4^.

Understanding the roles of PHYs in primary metabolism, especially carbohydrate metabolism, is of great importance in crop improvement practices under controlled light environments such as glasshouses^5^. Several “omics” studies, particularly transcriptomics, revealed that PhyA-mediated photomorphogenic responses involve global changes in gene expression^6^. In response to far-red and white light, PhyA regulates several primary metabolites, including amino acids, organic acids, and major sugars in Arabidopsis^7^. Subsequently, it was reported that loss of phytochrome impacts core metabolism and over-accumulation of a large number of primary metabolites have been observed in leaves of the Arabidopsis *phyBD* and *phy ABDE* mutants^8^.

Since proteins are directly involved in various cellular functions, a comprehensive proteomic investigation is likely to provide an insight into the intracellular changes as a result of the loss of photoreceptors. A few studies have reported the proteomics alterations in tomato seedling during the early stage of development, but their research was mainly focused either on proteome profiling of skotomorphogenesis or the changes in proteome during the transition from dark to red light. Therefore, the present study was undertaken to unravel the specific role of PhyA in regulating central metabolism during FR treatment using a high-throughput iTRAQ (isobaric tags for relative and absolute quantitation) based quantitative proteomics. Furthermore, GC–MS-based metabolite profiling was also performed to understand the underlying metabolite variation. In the present study, we also identified differentially expressed late response proteins in a longitudinal manner by taking a two-time point of data acquisition in every experiment. These proteins displayed a significantly altered expression and represented the foremost altered physiological pathways.

## Results and discussion

To facilitate photomorphogenesis, plants have evolved an array of photoreceptors, which capture a wide range of light spectrum and alter a myriad of physiological processes upon reception of the light signal^9,10^. Phytochromes that sense red (R) and far-red light (FR)^3^ are photoreceptors with which plants gather environmental information and play a critical role in growth and development. Following light exposure, signaling and transcription factor genes tend to be induced/repressed early, while genes in energy metabolism and photosynthesis were affected later. Metabolomics and gene expression studies of various species^6–8,11^ revealed the role of various photoreceptors in regulating primary metabolism in plants.

In the current study, we employed a proteomics-based analysis of tomato *fri* mutant grown under FR compared to Ailsa Craig (AC) seedlings as control, which has a functional Phytochrome A. Our results revealed key mechanistic insights for the understanding of functional mechanisms underlying in the process of FR light-mediated signal transduction. The majority of PhyA regulated genes displayed changes in their expression after several hours under FR, and these ‘late response genes’ represent genes involved in photosynthesis and chloroplast development. It is assumed that many downstream components are regulated after a longer duration of light treatment. The time points 48 and 96 h were taken into account to get a comprehensive insight into FR-mediated late response pathways.

In the present study, we first looked into the proteomic alteration as a result of FR irradiation in the wild-type AC seedlings. Subsequently, we checked the effect of loss of *PHYA* in FR-mediated signaling by studying the differential protein expression of *fri* mutant compared to AC. Additionally, we performed differential protein expression analysis of *fri* mutant and AC seedlings grown under dark to understand the specific role of phyA in the dark. To accomplish this, we performed integrated proteomics and metabolomics-based deep investigation. Mass spectrometry (MS) based quantitative proteomics has facilitated the identification of functional modules and pathways and prediction of biomarkers^12,13^. Quantitative results can be obtained using stable isotope labels or label-free methods^14–16^. Typically, isotope labels offer better reproducibility in quantitation than label-free methods as the latter require highly reproducible LC–MS/MS platforms^16^. The iTRAQ based data analysis strives to report reliable relative protein quantification; however, discrepancies in quantification might occur due to the low abundance of peptides, smaller sample size, complicated experimental procedures, and lack of suitable internal standards^17–19^. Notwithstanding several drawbacks, iTRAQ is one of the most flexible techniques that allow multiplexing of four, six, and eight separately labeled samples within one experiment, which ensures the reduction of costly LC–MS runtime^19–22^.

Metabolite profiling has also been considered as a powerful tool to examine the regulatory mechanisms of metabolic pathways in plants^23–25^. Although LC–MS is the most widely used platform for the analysis of intermediate molecules, from the past few decades, GC–MS has also been used to identify and quantify metabolites such as Calvin cycle pathway intermediates^26^. In the current study, proteomic and metabolite profiling data have been integrated to investigate the regulation of various biochemical pathways in tomato, which revealed a crucial relationship between signal transduction and metabolic pathways.

### Protein identification and quantification

**Figure 1:**
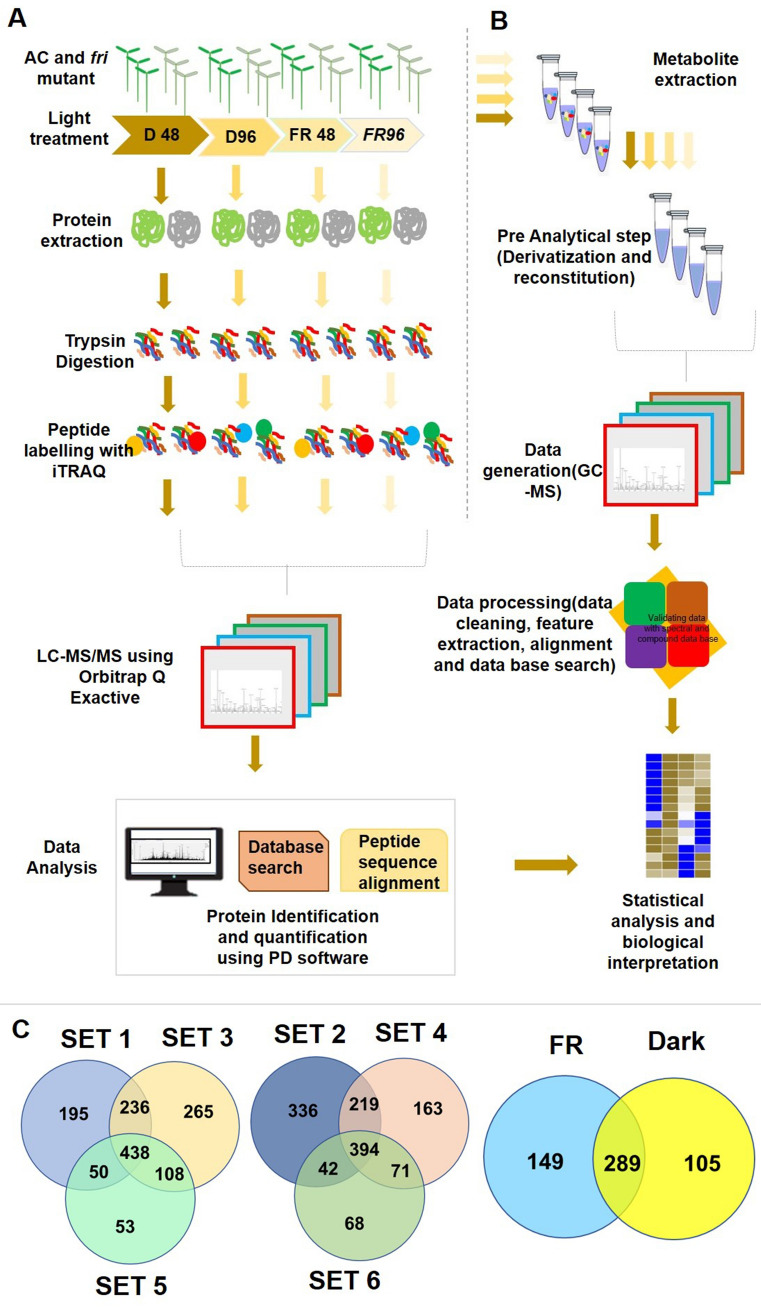
Quantitative proteomics and metabolite profiling of tomato seedlings to understand the role of PhyA in FR light-mediated photomorphogenesis. (**A**) Schematic illustration of the overall experimental strategy used in the discovery-phase global quantitative proteomics and metabolomics. (**B**) Venn diagram showing the unique and overlapping proteins identified in specific light treatment at two different time points.

In this study, we used Ailsa Craig (AC) and *fri (far-red insensitive)* mutant seedlings of tomato grown under FR light and dark. To understand the temporal variation in the expression, two-time points (48 and 96 h) of data acquisition were employed in every experiment (Fig. 1A). Three independent biological replicates of seedling samples were used for the iTRAQ experiment, and the detailed iTRAQ labeling strategy is shown in Supplementary Fig. S1. The iTRAQ reaction sets 1, 3, and 5 represent FR light-treated mutant and AC, while sets 2, 4, and 6 represent dark-grown seedlings. A total of 1519 proteins were identified in three biological replicates (Supplementary Table S1), and 289 proteins were found to be present in all the 3 replicates (Fig. 1C). 438 and 394 common proteins were found in FR and dark-grown samples, respectively (Fig. 1C). Raw abundance, normalized abundance, grouped abundance and abundance ratio of these proteins at various treatment conditions, is shown in Supplementary Table S1.

**Figure 2:**
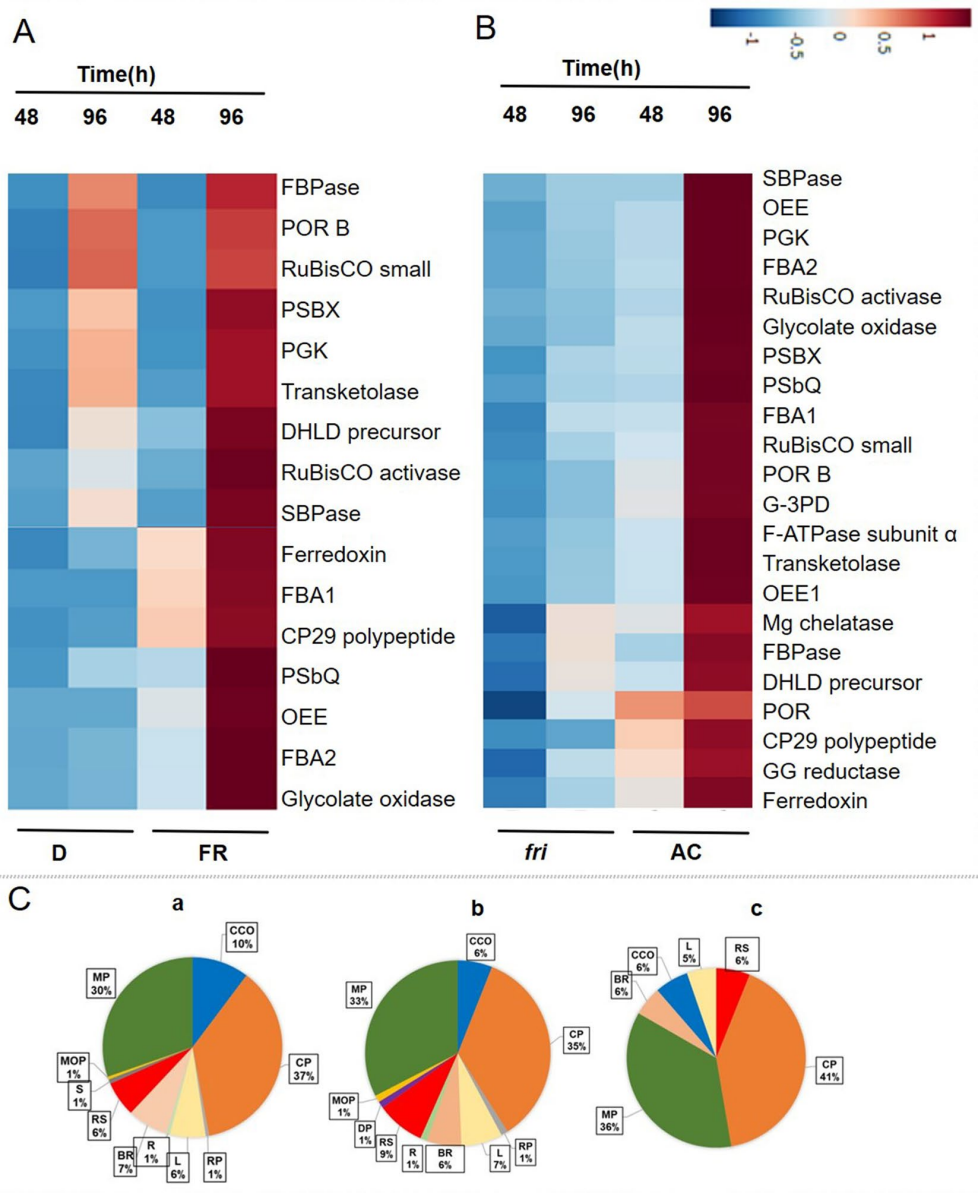
Quantitative proteomics to understand the role of PhyA in the light-mediated establishment of the photosynthetic machinery. (**A**) Heat map illustrating the fold ratio of differentially altered proteins in the central carbon metabolic pathways in response to FR irradiation in AC seedling in comparison to dark-grown AC control. (**B**) Heat map demonstrating the fold ratio of differentially altered proteins in the *fri* mutant in comparison to AC seedlings under FR irradiation. OEE1-33 kDa precursor protein of oxygen-evolving complex; OEE-The oxygen-evolving enhancer protein; DHLD precursor-dihydrolipoamide dehydrogenase precursor; RuBisco-ribulose-1,5bisphosphate carboxylase or oxygenase; PGK-phosphoglycerate kinase; G3PD: glyceraldehyde-3-phosphate dehydrogenase; SBPase- Sedoheptulose 1,7-bisphosphatase; Mgprot IX-Mg-protoporphyrin IX chelatase; GG reductase-Geranylgeranyl reductase; Por-light dependent NADH:protochlorophyllide oxidoreductase 1; PorB-light dependent NADH:protochlorophyllide oxidoreductase 3 s2; FBA1-Fructose-bisphosphate aldolase 1, FBA2-Fructose-bisphosphate aldolase; F ATPase subunit α-ATP synthase subunit alpha; PsbX-psbX photosystem II 23 kDa protein; PsbQ-photosystem II oxygen-evolving complex protein; (**C**) The GO terms identified for biological process (a) in all the identified proteins (b) significant proteins identified in the comparison of Dark vs FR in Ailsa craig (c) significant proteins identified in the comparison of Ailsa craig vs *fri* mutant grown under FR light. CCO (cellular component organization or biogenesis), CP (cellular process), RP (reproductive process), L (localization), BR (biological regulation), R (reproduction), RS (response to stimulus), DP (developmental process), MOP (multicellular organismal process), MP (metabolic process) and S (Signaling).

Gene ontology (GO) database, Panther classification system (http://www.pantherdb.org/)^27^ was used to categorize all of the identified proteins, and gene ontology (GO) analysis allowed us to understand the different functions and process in which the proteins are involved (Fig. 2C, Supplementary Fig. S2A and S2B). The molecular function (Supplementary Fig. S2A), cellular component (Supplementary Fig. S2B), and biological process Fig. 2C of the proteins are presented. According to the biological process analysis (Fig. 2C (a)), most of the proteins were related to cellular process (37%) and metabolic processes (30%). Most of the proteins were found in the Cell part (26%) or cell region (26%) as shown in Supplementary Fig. S2B (a) by cellular component analysis. Concerning Molecular function (Supplementary Fig. S2A (a)), the identified proteins were involved in catalytic activity (48%) and binding (34%). The identified proteins were filtered to verify whether the changes in protein abundance are significant based on the cutoff values with a fold change < 0.66 or > 1.5, and *p* value < 0.05. We performed our analysis in three categories: (1) differential protein expression analysis of AC seedlings irradiated with FR in comparison to dark control (AC/FR versus AC/D); (2) differential expression analysis of *PhyA* mutant (*fri*) seedlings irradiated with FR in comparison to AC seedlings (*fri*/FR versus AC/FR) to examine the role of light and the specific role of PhyA, respectively in regulating primary metabolism; and (3) differential expression analysis of *PhyA* mutant (*fri*) seedlings in comparison to AC seedlings under dark (*fri*/D vs AC/D) to examine the specific roles of Phy A in dark.

The statistically significant proteins (ANOVA passed *p* < 0.05) from both the groups AC/FR versus AC/D and *fri*/FR versus AC/FR were further subjected to PLS-DA (partial least squares discriminant analysis) using an online tool MetaboAnalyst 4.0^28^. Based on the differences among the protein fold ratio values on a 2D-score plot, the four groups that are segregated in AC/FR versus AC/D group analysis were ACD48, ACD96, ACFR48, and ACFR96 (Supplementary Fig. S3B). Similarly, MUTFR48, MUTFR 96, ACFR48, and ACFR96 were the 4 groups segregated in the analysis of *fri*/FR versus AC/FR (Supplementary Fig. S4B). Further, a heat map was generated demonstrating the differential expression (fold ratio pattern) of a few of these proteins involved in photosynthesis and carbon metabolism processes and is presented in Fig. 2A,B.

The differentially expressed proteins obtained in the iTRAQ study were integrated and highlighted in all possible KEGG (Kyoto Encyclopedia of Genes and Genomes) metabolic pathways using the KEGG Mapper tool^29^. Mapping of differentially expressed proteins indicated major perturbations in pathways associated with central metabolism. The coverage of the differentially expressed proteins was assessed using Plant-mPLoc (http://www.csbio.sjtu.edu.cn/bioinf/plant-multi/)^30^. Most of the proteins belonged to plastids, vacuoles, and mitochondria (Table 1).

**Table 1:** Proteins within each subcellular location group.

Sl.no	Accession no	Entry name	Subcellular compartment
1	Solyc10g006900.3.1	Light dependent NADH:protochlorophyllide oxidoreductase 3 s2	Chloroplast
2	Solyc07g044860.3.1	psbXphotosystem II 23 kDa protein	Chloroplast
3	Solyc07g032640.2.1	Oxygen-evolving enhancer protein 1	Chloroplast
4	Solyc02g065400.3.1	33 kDa precursor protein of oxygen-evolving complex	Chloroplast
5	Solyc02g079950.3.1	Photosystem II oxygen-evolving complex protein 3	Chloroplast
6	Solyc06g060340.3.1	Photosystem II subunit S	Chloroplast
7	Solyc03g115980.1.1	Geranylgeranyl reductase	Chloroplast
8	Solyc10g008740.3.1	Mg-protoporphyrin IX chelatase	Chloroplast
9	Solyc10g044520.2.1	Ferredoxin	Chloroplast
10	Solyc12g013710.2.1	Light dependent NADH:protochlorophyllide oxidoreductase 1	Chloroplast
11	Solyc02g086820.3.1	Chloroplast carbonic anhydrase	Chloroplast
12	Solyc07g066610.3.1	Phosphoglycerate kinase	Chloroplast
13	Solyc10g018300.2.1	Transketolase	Chloroplast
14	Solyc05g050970.3.1	Transketolase	Chloroplast
15	Solyc04g009030.3.1	Glyceraldehyde-3-phosphate dehydrogenase	Chloroplast
16	Solyc01g110360.3.1	Fructose-bisphosphate aldolase	Chloroplast
17	Solyc12g094640.2.1	Glyceraldehyde-3-phosphate dehydrogenase	Chloroplast
18	Solyc02g062340.3.1	Fructose-bisphosphate aldolase	Chloroplast
19	Solyc01g097460.3.1	Ribose 5-phosphate isomerase-related family protein	chloroplast
20	Solyc02g084440.3.1	Fructose-bisphosphate aldolase	Chloroplast
21	Solyc05g052600.3.1	Sedoheptulose-1,7-bisphosphatase	Chloroplast
22	Solyc12g009400.2.1	Pyruvate dehydrogenase E1 component subunit alpha	Chloroplast
23	Solyc02g091580.3.1	Oligopeptidase A	Chloroplast
24	Solyc05g050120.3.1	Cytosolic NADP-malic enzyme	Chloroplast
25	Solyc09g098120.3.1	Oil body-associated protein 1A	Chloroplast
26	Solyc03g118410.3.1	Acyl carrier protein	Chloroplast
27	Solyc04g073990.3.1	Annexin p34	Cytoplasm
28	Solyc09g090970.3.1	Major allergen Pruar 1	Cytoplasm
29	Solyc07g005390.3.1	Aldehyde dehydrogenase 11A3	Cytoplasm
30	Solyc01g087120.3.1	F1-ATP synthase delta subunit	Mitochondria
31	Solyc02g091560.3.1	Serine hydroxymethyltransferase	Mitochondria
32	Solyc12g042920.2.1	Cytochrome c1, heme protein	Mitochondria
33	Solyc08g065220.3.1	Glycine decarboxylase p-protein	Mitochondria
34	Solyc07g056540.3.1	Glycolate oxidase X92888	Peroxisome
35	Solyc09g025210.3.1	Alcohol dehydrogenase-2	Vacuole
36	Solyc09g072560.3.1	Legumin 11S-globulin	Vacuole
37	Solyc03g005580.2.1	11S storage globulin	Vacuole
38	Solyc09g090150.3.1	11S storage globulin	Vacuole
39	Solyc01g110110.3.1	Cysteine proteinase	Vacuole
40	Solyc09g065470.3.1	7S globulin	Vacuole
41	Solyc11g072380.2.1	Vicilin-like antimicrobial peptides 2–2	Vacuole
42	Solyc09g008770.3.1	Late embryogenesis abundant protein, putative	Cell wall
43	Solyc11g056680.1.1	Leucine-rich repeat receptor-like protein kinase family	Cell wall
44	Solyc01g074010.3.1	Protein kinase superfamily protein	Nucleus
45	Solyc04g074550.3.1	Cytochrome c oxidase subunit 6B	Nucleus
46	Solyc07g042440.3.1	Peroxiredoxin	Nucleus
47	Solyc01g074010.3.1	Protein kinase superfamily protein	Nucleus
48	Solyc01g080280.3.1	Chloroplast glutamine synthetase	Chloroplast, Mitochondrion
49	Solyc05g053300.3.1	Dihydrolipoamide dehydrogenase precursor	Chloroplast, mitochondrion
50	Solyc01g108600.3.1	Presequence protease 2	Chloroplast, mitochondrion
51	Solyc09g009390.3.1	Monodehydroascorbate reductase	Chloroplast, cytoplasm
52	Solyc10g082030.2.1	2-Cys peroxiredoxin 1	Chloroplast, cytoplasm
53	Solyc01g106430.3.1	Inorganic pyrophosphatase	Chloroplast, cytoplasm
54	Solyc02g080810.3.1	Aminomethyltransferase	Cytoplasm, mitochondrion
55	Solyc12g088670.2.1	Cysteine protease CYP1	Golgi apparatus, vacuole
56	Solyc02g079290.3.1	Protein SELF PRUNING 2G	Cytoplasm, nucleus
57	Solyc07g061790.3.1	SOUL heme-binding family protein	Chloroplast, Golgi apparatus

Accession number and description from the International Tomato Annotation Group (ITAG) release version 3.0.

### Temporal regulation of protein expression during the early development of AC seedlings

The proteome profile of FR-grown AC seedlings compared to dark-grown seedlings (AC/FR vs AC/D) revealed a total of 85 statistically significant proteins (Supplementary Fig. S3A) with high FDR confidence. Further, the selection was performed based on the fold ratio values (≥ 1.5 fold up-regulation or  ≤ 0.66 fold down-regulation), and the resulting proteins were considered for the subsequent pathway analysis. Comprehensive iTRAQ data analysis revealed a subset of 21 proteins present in both the time-points, while 13 and 21 different proteins were expressed exclusively in 48 and 96 h of FR-treatment, respectively. These FR-regulated proteins displaying differential expression patterns (Fig. 2A and Table 2) may provide vital clues to understand the proteome perturbations in response to light. The functional annotations revealed that several proteins involved in photosynthesis, carbon assimilation, and nitrogen metabolism (Table 2) exhibited a steep increase in accumulation, particularly with an increase in FR duration from 48 to 96 h. Interestingly, another set of proteins that are also involved in the establishment of photosynthetic machinery and carbon metabolism, showed an abundance independent of FR-treatment. However, the second category of proteins exhibited a temporal alteration in their accumulation profile in both FR-treated samples and dark controls. Our data suggest the presence of two distinct groups of proteins with either light-dependent or independent expression patterns (Fig. 2A). Most of the light-independent proteins showed a temporal variation with a significant change in their accumulation during 96 h of treatment (Fig. 2A and Table 2).

**Table 2:** List of differentially abundant proteins identified in tomato AC seedlings treated with FR light as a function of time.

Sl no	Acc.no	Entry name	Unique peptide	48 h (FR/D)		96 h (FR/D)	
				Mean	Std. error	Mean	Std. error
1	**Photosynthesis**						
2	Solyc10g006900.3.1	Light dependent NADH:protochlorophyllide oxidoreductase 3 s2	13	1.21	0.23	1.09	0.11
3	Solyc07g044860.3.1	psbXphotosystem II 23 kDa protein	8	1.04	0.18	1.52	0.17
4	Solyc07g032640.2.1	Oxygen-evolving enhancer protein 1	9	NS	NS	NS	NS
5	Solyc02g079950.3.1	Photosystem II oxygen-evolving complex protein 3	5	NS	NS	NS	NS
6	Solyc06g060340.3.1	Photosystem II subunit S	3	NS	NS	NS	NS
7	Solyc06g063370.3.1	Type I (26 kD) CP29 polypeptide	3	NS	NS	NS	NS
8	Solyc10g044520.2.1	Ferredoxin	1	NS	NS	NS	NS
9	Solyc02g083810.3.1	Ferredoxin–NADP reductase	3	NS	NS	NS	NS
10	Solyc01g079470.3.1	CP12	1	NS	NS	NS	NS
	**Carbon metabolism**						
11	Solyc05g050970.3.1	Transketolase	10	1.21	0.19	1.24	0.14
12	Solyc04g009030.3.1	Glyceraldehyde-3-phosphate dehydrogenase	8	NS	NS	NS	NS
13	Solyc01g110360.3.1	Fructose-bisphosphate aldolase	9	NS	NS	NS	NS
14	Solyc02g063150.3.1	RuBP carboxylase small subunit	6	1.17	0.13	1.07	0.24
15	Solyc12g094640.2.1	Glyceraldehyde-3-phosphate dehydrogenase	7	NS	NS	NS	NS
16	Solyc02g062340.3.1	Fructose-bisphosphate aldolase	4	NS	NS	NS	NS
17	Solyc02g084440.3.1	Fructose-bisphosphate aldolase	8	NS	NS	NS	NS
18	Solyc02g086820.3.1	Chloroplast carbonic anhydrase	5	NS	NS	NS	NS
19	Solyc07g056540.3.1	Glycolate oxidase X92888	6	NS	NS	NS	NS
20	Solyc01g106430.3.1	Inorganic pyrophosphatase	3	1.52	0.08	1.11	0.17
22	Solyc01g080460.3.1	Pyruvate orthophosphate dikinase	9	1.23	0.12	1.06	0.02
23	Solyc11g009080.2.1	DAHP synthase 1 precursor	3	NS	NS	NS	NS
	**Nitrogen assimilation, amino acid biosynthesis and protein degradation**						
24	Solyc02g080810.3.1	Amino methyltransferase	8	1.16	0.04	1.55	0.25
25	Solyc01g080280.3.1	Chloroplast glutamine synthetase	6	NS	NS	NS	NS
26	Solyc02g091580.3.1	Oligopeptidase A	5	NS	NS	NS	NS
	**Signaling**						
27	Solyc07g061790.3.1	SOUL heme-binding family protein	3	NS	NS	NS	NS
	**Storage**						
28	Solyc03g005580.2.1	11S storage globulin	35	0.96	0.05	0.62	0.05
29	Solyc11g072380.2.1	Vicilin-like antimicrobial peptides 2–2	11	1.00	0.03	0.51	0.07
30	Solyc01g090360.3.1	Non-specific lipid-transfer protein	8	0.64	0.10	1.02	0.22
31	Solyc10g075050.2.1	Non-specific lipid-transfer protein	6	0.58	0.11	0.55	0.19
32	Solyc02g077430.3.1	Phospholipase A1	5	1.40	0.22	0.49	0.02
33	Solyc04g007570.2.1	GDSL esterase/lipase 6	1	NS	NS	NS	NS
	**Stress and defence**						
34	Solyc12g094620.2.1	Catalase	14	0.92	0.14	0.81	0.05
35	Solyc12g010820.2.1	Late embryogenesis abundant protein-like	4	NS	NS	NS	NS
36	Solyc01g079820.3.1	Peroxiredoxin	4	1.27	0.17	1.05	0.09

A representative list of proteins from the iTRAQ data (Mean) showing a differential expression pattern. Accession number and description from the International Tomato Annotation Group (ITAG) release version 3.0.

*Light-dependent protein* proteins such as Cp29, Ferredoxin, PSII oxygen-evolving complex protein 3(PSbQ), Oxygen-evolving enhancer protein 1(OEE), and Fructose-bisphosphate aldolase (FBA 1 and 2), are some of the prominent proteins associated with photosynthesis and carbon assimilation and exhibited a light-dependent expression. Consistent with the acquisition of photoautotrophy during de-etiolation, an increase in their abundance was perceptible at 48 h followed by a steep increase in abundance at 96 h. Glycolate oxidase, one of the key proteins involved in photorespiration as well as mobilization of the stored reserve, too showed increased abundance with longer FR-irradiation (Fig. 2A).

*Light independent protein* Interestingly, PsbX (psbX photosystem II 23 kDa protein), Rubisco activase, RUBISCO small subunit, etc., known for their important involvement in de-etiolation, exhibited a light-independent accumulation. Consistent with the need to sustain the energy need of the growing seedlings, the proteins associated with energy metabolism viz. phosphoglycerate kinase (PGK), transketolase, dihydrolipoamide dehydrogenase precursor (DHLD precursor), sedoheptulose-1,7-bisphosphatase (SBPase), fructose-1,6-bisphosphatase (FBPase), showed increased accumulation at 96 h than at 48 h (Fig. 2A). Interestingly, our study showed a light-independent, temporal increase in the abundance of POR B (Solyc10g006900.3.1), a prominent protein involved in chlorophyll synthesis (Fig. 2A and Table 2).

PhyA is unique within the phytochrome family, as it is solely responsible for seedling responsiveness to continuous far-red light (FR)^31,32^. The de-etiolation process that occurs exclusively under FR^31^ provides an ideal opportunity to define the relationship between PhyA and its target gene ensemble. In Arabidopsis, gene expression profiling studies revealed that under FR irradiation, PhyA-regulated gene expression can be classified into ‘early-response’ genes and that accounts for 8% of total PhyA regulated expression^6^ and the late response genes whose expression manifest after several hours under FR.

Our data provides clues regarding the change in metabolism or regulation of protein levels during the transition from heterotrophic to autotrophic development. During heterotrophic-to-autotrophic transition, chloroplast development and differentiation are modulated by external light parameters and homeostasis of several plant hormones. These processes also include the regulation of gene expression, metabolites levels such as sugars, and reactive oxygen species^33^. Our results showed that the majority of proteins begin to accumulate after 48 h and reach their maximum response within 96 h, which indicates that the major part of the cellular functions underlying the de-etiolation process is established between 48 to 96 h of the FR treatment. This pattern is most striking for the most abundant classes of late response proteins, especially proteins that are associated with photosynthesis/chloroplast and cellular metabolism.

Light-mediated photomorphogenesis is often coupled with modulation of gene expression leading to various morphological changes. Plants possess complex networks for the maintenance of metabolic balance, particularly primary metabolism, and are accompanied by changes in mRNA levels, protein accumulation, enzyme activities, and metabolite levels. In a dark-grown seedling, the photomorphogenic developmental pathway is repressed and has a very different developmental pattern, which is known as skotomorphogenesis^34^. In skotomorphogenesis, resources are allocated towards hypocotyl elongation at the expense of cotyledon and root development. This growth strategy assures that limited seed reserves are used cautiously until they find sunlight which is a pre-requisite for photoautotroph survival^35^.

However, our study showed that a set of proteins exhibited a limited difference in abundance between FR and dark treatment. Light independent proteins that showed no change in abundance in response to light treatment suggest that large gene expression changes are not necessarily reflected in large protein abundance changes and these proteins are developmentally regulated on a temporal scale.

It has previously been shown that in Angiosperms photoconversion of protochlorophyllide (Pchlide) to chlorophyllide (Chlide) by the enzyme NADPH: Pchlide oxidoreductase (POR) is inefficient under FR wavelengths, and seedlings grown under prolonged FR fail to accumulate chlorophyll^36,37^. The phenomenon, also known as the FR block of greening response, has been characterized as a reduction of POR A (and partially of POR B) and associated damage of the membrane system of the prolamellar body^38,39^. However, Runge et al.^39^ reported that POR A expression rapidly becomes undetectable after illumination under continuous FR, whereas POR B expression persists throughout the greening process. Their study suggests that the two enzymes perform a unique biological function during development. In line with these observations, our results showed that there is an accumulation of POR B even in the FR-treated seedlings. Our results also reveal that FR treatment does not influence POR B accumulation, as both dark- and FR-treated seedlings showed similar levels at 48 h, but there is an increased protein abundance at 96 h in both the treatment. The higher abundance of POR B at 96 h indicates that as under FR there is no conversion of PChl to Chl, the turnover of POR B protein is not initiated under de-etiolation under FR.

### Loss of Phytochrome A results in the decreased abundance of proteins associated with primary metabolism

Being sessile organisms, plants must adjust their growth and development to the ambient light environment. Variations in light quality in the red and far-red regions of the spectrum (i.e. R: FR ratio) are sensed by the phytochromes. In response to low R: FR ratio signals, many plants exhibit shade avoidance syndrome, in anticipation of being shaded^40^. This process is often executed at the expense of leaf and storage organ development. Shade-avoidance syndrome provides an essential survival strategy in rapidly growing populations. Although shade avoidance may have major fitness benefits in crowded communities, the reallocation of resources towards elongation growth may lead to an increased risk of lodging and mechanical injury^41^. The roles of individual phytochromes in facilitating responses to low R: FR ratios have been mostly deduced from studies using mutants deficient in one or more family members. Many of these studies have confirmed the key involvement of phyB in transducing the low R: FR ratio signal, and inducing shade avoidance response^42^.

In contrast to other phytochromes, phyA is a light labile phytochrome, which subjects to rapid proteolytic degradation upon photoconversion to Pfr and accumulates to high levels only in etiolated seedlings^43^. Despite being present at reduced levels in light-grown plants, phyA performs an important role in the regulation of hypocotyl elongation in response to shade (reduced R: FR ratio). The enhanced hypocotyl elongation in *phyA* mutant seedlings under low R: FR ratio light^44^, led to the suggestion that in wild-type plants, phyA action was antagonizing phyB-mediated shade avoidance by constraining hypocotyl extension. The action of phyA in constraining shade-avoidance elongation responses has been shown to be highly essential in the seedling establishment under dense natural vegetational shade^45^. The growth-inhibitory action of phyA in low R: FR ratio conditions can be successfully exploited to eliminate the unwanted elongation responses in more densely planted crops^46^.

To examine the role of PhyA in regulating primary metabolism, *fri* mutant seedlings were grown under continuous FR along with AC seedlings (Fig. 1A). FR light insensitive (*fri*) mutant of tomato harbors a base substitution in *PHYA* gene resulting in aberrant processing of the pre-mRNA^47^. The physiological characterization of the mutant seedlings revealed that they are insensitive to FR light and they exhibit loss of FR-HIR for hypocotyls inhibition. The seedlings were treated for 48 h and 96 h under FR to study the temporal regulation of protein. It is known that under continuous FR, PhyA is the prominent phytochrome molecule involved in the de-etiolation process, and loss of *PHYA* gene function might lead to altered protein accumulation^37,48^. A total of 135 statistically significant proteins were identified (Supplementary Fig. S4A) from the iTRAQ-based quantitative temporal proteomic analysis of *fri* mutant grown under FR (*fri*/FR versus AC/FR). Our analysis revealed a subset of 69 proteins common to both the time-points, while 22 and19 different proteins were expressed exclusively in 48 and 96 h of treatment, respectively. When we examined the protein expression profile of FR treated *fri* mutant, we found a significant proteome alteration associated with photosynthesis, electron transfer, and carbon and nitrogen metabolism. Proteins associated with chlorophyll synthesis, ATP production, Calvin cycle, glycolysis, and TCA cycle (Fig. 2B) are severely affected in the *fri* mutant and showed a drastically decreased abundance on longer treatments (96 h) compared to the control sample. Interestingly, light-independent proteins, which are discussed in the previous session, also showed a decreased abundance in the *fri* mutant (Fig. 2B and Table 3).

**Table 3:** List of differentially expressed proteins in *fri* mutant at different time points.

Sl.No	Acc.no	Entry name	Unique peptide	48 h (MUT/AC)		96 h (MUT/AC)	
				Mean	Std. error	Mean	Std. error
	**Photosynthesis**						
1	Solyc10g006900.3.1	Light dependent NADH:protochlorophyllide oxidoreductase 3 s2	5	0.25	0.04	0.33	0.05
2	Solyc07g044860.3.1	psbXphotosystem II 23 kDa protein	8	0.28	0.04	0.31	0.06
3	Solyc12g013710.2.1	Oxygen-evolving enhancer protein 1	4	0.70	0.15	0.33	0.01
4	Solyc07g032640.2.1	Oxygen-evolving enhancer protein	2	0.24	0.02	0.42	0.07
5	Solyc02g065400.3.1	33 kDa precursor protein of oxygen-evolving complex	2	0.25	0.03	0.34	0.05
6	Solyc02g079950.3.1	Photosystem II oxygen-evolving complex protein 3	5	0.24	0.02	0.33	0.10
7	Solyc06g060340.3.1	Photosystem II subunit S	3	0.25	0.03	0.40	0.14
8	Solyc06g063370.3.1	Type I (26 kD) CP29 polypeptide	3	0.17	0.04	0.20	0.05
9	Solyc06g072540.1.1	ATP synthase subunit alpha, chloroplastic	2	0.28	0.02	0.46	0.09
10	Solyc01g087120.3.1	F1-ATP synthase delta subunit	4	0.85	0.11	0.60	0.02
11	Solyc03g115980.1.1	Geranylgeranyl reductase	4	0.52	0.04	0.41	0.10
12	Solyc10g084040.2.1	Thylakoid lumenal 15.0 kDa protein 2, chloroplastic	3	0.44	0.04	0.59	0.08
13	Solyc10g008740.3.1	Mg-protoporphyrin IX chelatase	4	0.65	0.05	0.43	0.11
14	Solyc10g044520.2.1	Ferredoxin	1	0.39	0.01	0.33	0.02
	**Carbon metabolism**						
15	Solyc07g066610.3.1	Phosphoglycerate kinase	14	0.25	0.03	0.50	0.07
16	Solyc10g086580.2.1	Ribulose bisphosphate carboxylase/oxygenase activase	15	0.12	0.01	0.30	0.05
17	Solyc10g018300.2.1	Transketolase	8	0.27	0.01	0.43	0.08
18	Solyc05g050970.3.1	Transketolase	8	0.50	0.05	0.68	0.14
19	Solyc04g009030.3.1	Glyceraldehyde-3-phosphate dehydrogenase	3	0.23	0.01	0.27	0.04
20	Solyc01g110360.3.1	Fructose-bisphosphate aldolase	5	0.46	0.11	0.56	0.15
21	Solyc05g053300.3.1	Dihydrolipoamide dehydrogenase precursor	6	0.67	0.06	0.61	0.08
22	Solyc02g063150.3.1	RuBP carboxylase small subunit	2	0.34	0.07	0.43	0.18
23	Solyc12g094640.2.1	Glyceraldehyde-3-phosphate dehydrogenase	5	0.17	0.03	0.41	0.06
24	Solyc02g062340.3.1	Fructose-bisphosphate aldolase	2	0.18	0.01	0.33	0.02
25	Solyc01g097460.3.1	Ribose 5-phosphate isomerase-related family protein	4	0.43	0.04	0.51	0.10
26	Solyc02g084440.3.1	Fructose-bisphosphate aldolase	5	0.33	0.02	0.40	0.10
27	Solyc02g086820.3.1	Chloroplast carbonic anhydrase	5	0.16	0.01	0.56	0.21
28	Solyc07g056540.3.1	Glycolate oxidase X92888	6	0.15	0.02	0.33	0.04
29	Solyc05g050120.3.1	Cytosolic NADP-malic enzyme	5	0.88	0.09	0.62	0.14
30	Solyc05g052600.3.1	Sedoheptulose-1,7-bisphosphatase	4	0.17	0.02	0.40	0.06
31	Solyc12g009400.2.1	Pyruvate dehydrogenase E1 component subunit alpha	3	0.55	0.02	0.67	0.10
32	Solyc01g106430.3.1	Inorganic pyrophosphatase	3	0.66	0.05	0.50	0.07
33	Solyc07g005390.3.1	aldehyde dehydrogenase 11A3	2	0.38	0.04	0.51	0.04
34	Solyc10g047430.1.1	LOW QUALITY:Ribulose bisphosphate carboxylase large chain	2	0.38	0.06	0.36	0.03
	**Nitrogen assimilation, amino acid biosynthesis and protein degradation**						
35	Solyc02g091560.3.1	Serine hydroxymethyltransferase	9	0.17	0.01	0.33	0.08
36	Solyc08g065220.3.1	Glycine decarboxylase p-protein	11	0.33	0.06	0.56	0.04
37	Solyc01g009990.3.1	Peptidyl-prolyl cis–trans isomerase	5	0.72	0.12	0.45	0.10
38	Solyc02g080810.3.1	Aminomethyltransferase	8	0.39	0.01	0.55	0.09
39	Solyc01g080280.3.1	Chloroplast glutamine synthetase	6	0.37	0.05	0.34	0.01
40	Solyc01g108600.3.1	Presequence protease 2	6	0.64	0.03	0.53	0.08
41	Solyc12g088670.2.1	Cysteine protease CYP1	4	0.37	0.04	0.38	0.06
42	Solyc02g091580.3.1	Oligopeptidase A	5	0.57	0.07	0.74	0.05
43	Solyc01g110110.3.1	Cysteine proteinase	4	2.19	0.53	0.65	0.04
	**Stress and defense**						
44	Solyc09g008770.3.1	Late embryogenesis abundant protein, putative	18	1.58	0.22	1.09	0.17
45	Solyc09g090970.3.1	Major allergen Pruar 1	10	1.58	0.43	1.71	0.21
46	Solyc09g009390.3.1	Monodehydroascorbate reductase	8	0.56	0.05	0.64	0.09
47	Solyc10g082030.2.1	2-Cys peroxiredoxin 1	3	0.55	0.03	0.64	0.03
48	Solyc04g073990.3.1	Annexin p34	10	1.94	0.28	1.44	0.23
49	Solyc06g048840.3.1	Late embryogenesis abundant protein	7	1.45	0.57	0.46	0.04
50	Solyc07g042440.3.1	Peroxiredoxin	5	0.57	0.03	0.53	0.06
	**Signalling**						
51	Solyc11g056680.1.1	Leucine-rich repeat receptor-like protein kinase family	4	0.57	0.09	0.45	0.01
52	Solyc01g074010.3.1	Protein kinase superfamily protein	1	0.47	0.08	0.73	0.16
53	Solyc07g061790.3.1	SOUL heme-binding family protein	3	0.27	0.02	0.31	0.05
	**Storage**						
54	Solyc09g025210.3.1	Alcohol dehydrogenase-2	35	2.27	0.26	1.24	0.07
55	Solyc09g072560.3.1	Legumin 11S-globulin	25	2.63	0.88	1.19	0.07
56	Solyc03g005580.2.1	11S storage globulin	34	2.09	0.38	1.17	0.08
57	Solyc09g090150.3.1	11S storage globulin	23	2.12	0.37	1.58	0.25
58	Solyc09g065470.3.1	7S globulin	14	3.66	1.19	0.99	0.18
59	Solyc11g072380.2.1	Vicilin-like antimicrobial peptides 2–2	11	2.52	0.51	1.09	0.14
60	Solyc09g098120.3.1	Oil body-associated protein 1A	5	1.64	0.21	1.05	0.03
61	Solyc03g118410.3.1	Acyl carrier protein	2	0.24	0.01	0.35	0.03
	**Meristem development/branching**						
62	Solyc02g079290.3.1	Protein SELF PRUNING 2G	3	1.84	0.33	0.85	0.12
	**Hormone signaling (GA)**						
63	Solyc01g006580.3.1	2-oxoglutarate-dependent dioxygenase-related family protein	3	0.58	0.09	0.38	0.03

A representative list of proteins from the iTRAQ data (Mean) showing a differential expression pattern. Accession number and description from the International Tomato Annotation Group (ITAG) release version 3.0.

Collectively, global proteome analysis provides support for several generalizations. The data confirms that most of the proteins exhibit significant responses to FR in the control relative to *fri* mutant. Thus, these data furnish strong verification that the genes identified here are regulated by PhyA in response to the FR light signal. Moreover, our results showed that the majority of genes begin to respond to the light signal after 48 h and reach their maximum response within 96 h. The results confirm that the major part of the cellular functions underlying the de-etiolation process is established after 48 h of the FR treatment.

The global proteomics analysis revealed alterations among various protein groups and for a better understanding of their functions, they have been categorized into small sub-groups, which are discussed in detail hereafter.

*Photosynthesis light reaction* The loss of PhyA resulted in an overall decrease in the abundance of photosynthetic proteins, which could be due to the involvement of PhyA in the biosynthesis of chlorophyll and other associated proteins. The development of chloroplasts and their preparation for the photosynthetic function is regarded as some of the prominent features of the seedling establishment during early development. Several studies have revealed that Phys are important regulators of the synthesis of photosynthetic pigment^49^. The R-light treatment has been shown to induce the chlorophyll synthesis^50^, while depletion of phytochromes in the R-grown seedlings leads to concomitant reductions in chlorophyll levels^51^.

Proteins like light-dependent NADH: protochlorophyllide oxidoreductase 1 or Por isoform (Solyc12g013710.2.1), light-dependent NADH: protochlorophyllide oxidoreductase 3 or PorB (Solyc10g006900.3.1), Geranylgeranyl reductase (Solyc03g115980.1.1), and Mg-protoporphyrin IX chelatase (Solyc10g008740.3.1), which are involved in chlorophyll biosynthetic process, exhibited a decreased abundance in the mutant (Fig. 2B). The light-dependent reaction is performed by several intrinsic membrane-protein complexes named photosystem I (PSI), photosystem II (PSII), cytochrome (cyt) b6f. complex, and ATP synthase^52^. Several proteins that are identified to be the components of the reaction center of PS-II (photosystem-II), Oxygen evolving complex, ATP synthase subunit, etc. showed a decrease in their accumulation in the mutant as compared to their control when treated under FR (Fig. 2B).

*Carbon assimilation and carbohydrate metabolism* Proteins involved in carbon assimilation and carbohydrate metabolism showed a decreased abundance in the mutant grown under FR when compared to AC seedlings grown under the same condition. Rubisco (large and small subunits) and Rubisco activase exhibited a decreased abundance in the *fri* mutant compared to AC seedlings treated under FR. The chloroplast enzyme sedoheptulose 1,7-bisphosphatase (SBPASE), a key enzyme in the Calvin cycle, catalyzes the dephosphorylation of sedoheptulose-1,7-bisphosphate to sedoheptulose-7-phosphate and also involved in the regulation of the carbon flow ^53–55^, showed a decrease in abundance in the mutant under continuous FR treatment when compared to AC seedlings. Other Calvin cycle enzymes like phosphoglycerate kinase and Fructose 1.6-bisphosphate aldolase (Solyc02g062340.3.1) also exhibited a decreased abundance when treated under FR light compared to the control seedlings (Fig. 2B and Table 3).

The FR-grown mutant seedlings displayed a reduced plastidial glycolysis pathway with a reduced abundance of glycolytic enzymes such as Fructose 1,6-bisphosphate aldolase (Solyc02g084440.3.1), phosphoglycerate kinase catalyzes the phosphorylation of 3-phosphoglycerate to 1,3-diphosphoglycerate, and Glyceraldehyde-3-P dehydrogenase (GAPC) compared to the control seedlings grown under the same condition. Glyceraldehyde-3-P dehydrogenase, a key enzyme in energy metabolism, mediates the conversion of glyceraldehyde-3-P to 1,3-bis-phosphoglycerate, found to be involved in several cellular functions apart from glycolysis.

In our study pyruvate dehydrogenase E1 component subunit alpha (Solyc12g009400.2.1), showed a decline in their abundance in the mutant seedlings compared to the control seedlings when grown under FR. Proteins of pyruvate dehydrogenase complex, including dihydrolipoyl dehydrogenase and dihydrolipoamide dehydrogenase precursor too, exhibited a decreased level of abundance in the mutant seedlings grown under FR light compared to their corresponding controls (Fig. 2B and Table 3). The loss of phytochrome results in retarded growth, and in such cases, resources are allocated towards resilience than growth, and this strategy is based on changing the metabolism at a fundamental level^8,11^. Yang et al*.*^8^ performed comprehensive metabolomics and gene expression studies of *Arabidopsis thaliana* to determine the role of Phytochrome in central metabolism. The study revealed that loss of phytochrome impacts core metabolism. Han et al.^56^ reported that PhyA and PhyB signaling play important roles in the regulation of carbon metabolism in plant leaves, being influenced not only by the quantity of light but also the quality. Carlson et al*.*^57^ investigated the transcriptomic and gel-based proteomic profile of tomato seedlings during the transition from dark to light (red light) and found that PhyA regulates carbon flux through major primary metabolic pathways. A study by Fox et al*.*^58^ has revealed the contribution of various photoreceptors in the regulation of proteins involved in chloroplast metabolism and the Calvin cycle, including Rubisco and Rubisco activase. The quadruple *phyA;phyB;cry1;cry2* mutant exhibited a reduced CO_2_ fixation and impaired activity of the Calvin cycle proteins with decreased levels of chlorophyll. These data suggest that Phy and Cry signaling has a crucial role in photosynthesis and carbon fixation using the Calvin cycle. Our data indicate that PhyA action is important in regulating carbon assimilation, and loss of the photoreceptors results in a concomitant decrease in the carbon assimilation efficiency and alteration in carbon flux.

**Figure 3:**
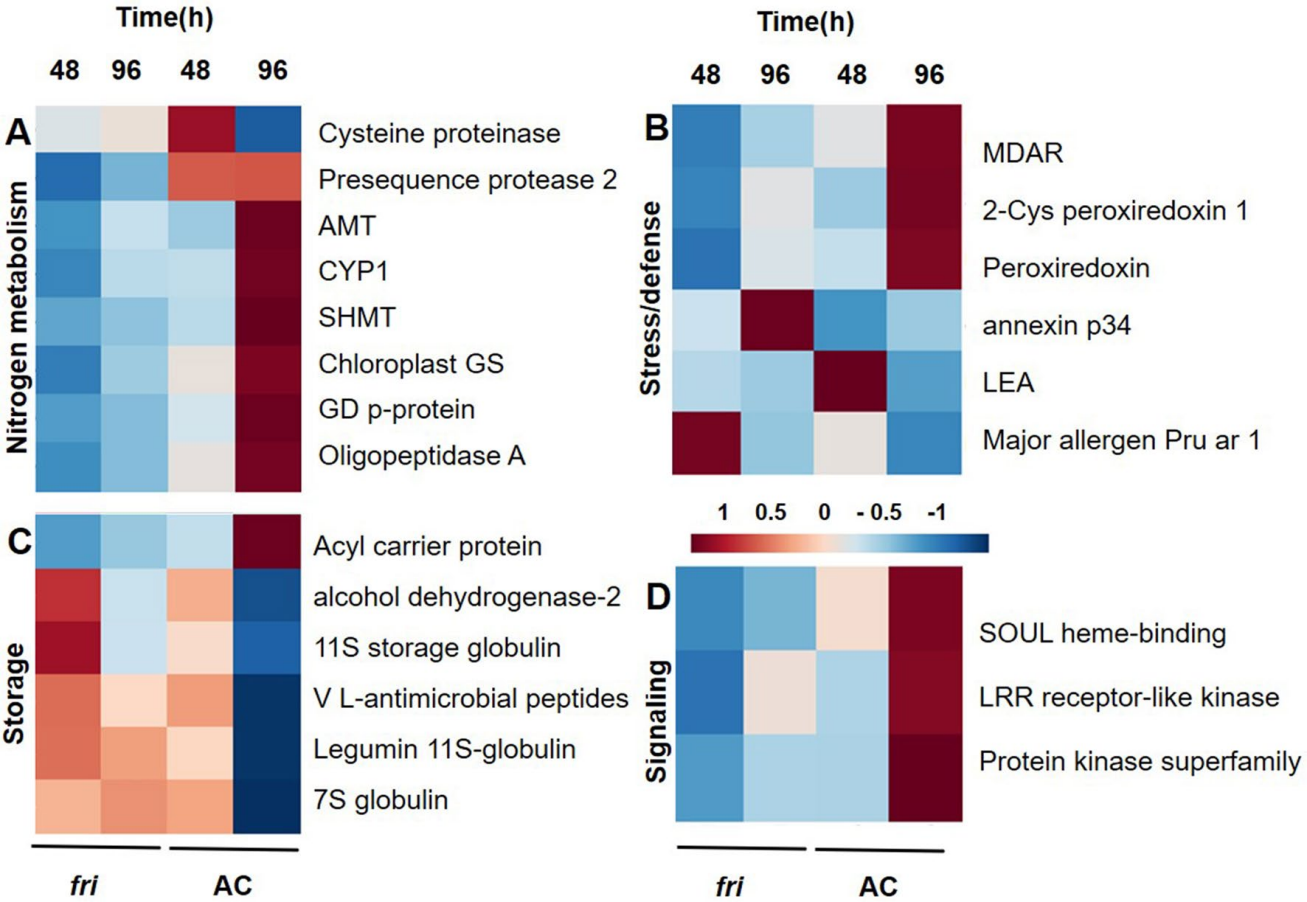
PhyA regulated proteins associated with nitrogen metabolism, storage, and stress/defense metabolic pathways. Heat map showing the fold ratio of differentially altered proteins involved in nitrogen metabolism (**A**), stress/defense (**B**) storage (**C**), and signaling (**D**) metabolic pathways in *fri* mutant in comparison to AC seedlings under FR irradiation. MDAR-Monodehydroascorbate reductase; Chloroplast GS-chloroplast glutamine synthetase; AMT-Amino methyltransferase; SHMT-Serine hydroxymethyltransferase; GD p-protein-glycine decarboxylase p-protein; cysteine protease CYP1; LEA-Late embryogenesis abundant protein; LRR-Leucine-rich repeat.

*Amino acid biosynthesis and protein degradation* The photosynthetically assimilated carbon is distributed into different metabolic pathways to either provide energy for growth and development or to synthesize amino acids for protein synthesis. Evidence suggests that phytochrome signaling has sizable effects on nitrogen metabolism^7,50,56^. Loss of PhyA resulted in an alteration in the nitrogen metabolism and both amino acid biosynthesis and protein degradation processes were de-regulated in mutant seedling grown under FR. Overall, eight proteins associated with nitrogen metabolism were dysregulated in the mutant. Among these proteins, chloroplast glutamine synthetase (GS) (Solyc01g080280.3.1), Serine hydroxymethyltransferase (SHMT) (Solyc02g091560.3.1), glycine decarboxylase p-protein (GDC) (Solyc08g065220.3.1), and amino methyltransferase (Solyc02g080810.3.1) displayed a decrease in abundance compared to the control seedling when treated under FR (Fig. 3 and Table 3). GS is a key assimilatory enzyme for ammonia^59^, primarily responsible for scavenging ammonia, a highly reactive and cytotoxic metabolite, and converts it to glutamine^60^. SHMTs, along with GDC (glycine decarboxylase complex), being involved in the transfer of hydroxymethyl of serine to typically polyglutamylated tetrahydrofolate producing Gly and 5,10-methylene THF^61^, while amino methyltransferase is involved in the glycine catabolism.

Protein proteolysis is indispensable for many plant signal transduction pathways and regulates the developmental stages of a plant by removing harmful or damaged proteins and supply cells with amino acids^62^. Proteases are thus the major players in the maintenance of cell homeostasis. Additionally, proteases also perform a regulatory role in a variety of processes that are essential for growth, development, reproduction, immune response, embryogenesis, photosynthesis, programmed cell death (PCD), etc^63^. Our results showed dysregulation of several proteases in the mutant grown under FR compared to the control. Cysteine proteinase (Solyc01g110110.3.1) showed a decrease in its accumulation in the mutant during early (48 h) treatment under FR when compared to the wild type at this time point, but continuous treatment in FR resulted in a 1.9-fold increase in the abundance. However, other proteases such as cysteine protease CYP1(Solyc12g088670.2.1), presequence protease 2 (Solyc01g108600.3.1), and oligopeptidase A (Solyc02g091580.3.1) showed diminished accumulation in the mutant in both the time points of treatment compared to the control seedlings. Peptidyl-prolyl cis–trans isomerase (Solyc01g009990.3.1) which is involved in protein folding also showed decrease in its abundance ^64^ compared to control seedlings when treated under FR (Fig. 3, and Table 3). As carbon metabolism was found to be severely affected in the mutant, there could be a possibility of inadequate reduced carbon in the cell. This could be one of the reasons why most of the proteins associated with nitrogen metabolism showed a decreased abundance in the mutant. However, the exact role of PhyA in regulating nitrogen metabolism needs to be investigated further.

*Storage protein* During seed germination, storage proteins are degraded and act as a source of amino acid, while storage lipids are mobilized to deliver an energy source for the developing seedlings^23,65^. Developing seedlings allocate their nutritional reserves, which include storage proteins and lipids, for hypocotyl extension, and develop machinery for photosynthesis. Among the 8 differentially expressed proteins, all the proteins were found to exhibit an increased accumulation in the mutant when compared to the control seedlings. Previous studies revealed that globulin mobilization becomes evident only after 48–72 h imbibition^66^. Globulins are broken down in the protein bodies by proteinases that are synthesized in RER (rough endoplasmic reticulum). In our study, seed storage proteins like 7S globulin (Solyc09g065470.3.1) and 11S Globulins (Solyc03g005580.2.1, Solyc09g090150.3.1) were found to be accumulated in the *phyA* mutant after 96 h of FR treatment when compared to the control seedlings at this time point. Similarly, vicilins (Solyc09g082340.2.1, Solyc11g072380.2.1) and legumin (Solyc09g072560.3.1) that are shown to be expressed in developing seedlings showed a similar trend. Oil body-associated protein (Solyc11g072380.2.1), is storage associated protein involved in accumulating nutrients and lipid; and protein trafficking between organelles^67^, which also showed an increase in abundance compared to the control seedlings (Fig. 3 and Table 3).

The data suggests a slow breakdown of storage protein in the *fri* mutant^57^ and it could be an indication of the absence of light-dependent development in phytochrome mutant as PhyA is the primary photoreceptor responsible for perceiving and mediating various responses to FR light^68–70^. In control seedling, most of the sugars are consumed after 96 h, under FR. As *phyA* mutant under FR exhibits skotomorphogenesis, there is a possible conversion of sugars into storage material instead of converting into fatty acids as in photomorphogenesis^50^. The decreased abundance of an acyl carrier protein (Solyc03g118410.3.1) compared to the control seedlings, a major protein involved in fatty acid synthesis^71^, suggests an absence of sugar conversion in the mutant.

*Stress and defense* An appropriate light environment is a critical requirement for the establishment of proper resistance responses in defense responses^72^. Several defense and stress responses in plant species like rice and Arabidopsis were identified as light-dependent^73–76^, and studies suggest that light could regulate defense response either through light-driven chemical reaction or through downstream light-responsive signaling pathways^72^. Previous microarray studies^6,77^ demonstrated that the expression of many plant defense genes is controlled by the activity of phytochromes.

In the present study, seven stress and defense-related proteins were differentially expressed in *fri* mutant seedlings compared to control seedlings, including Late embryogenesis abundant protein, putative (Solyc09g008770.3.1), annexin p34 (Solyc04g073990.3.1), Major allergen Pruar 1 (Solyc09g090970.3.1), Monodehydroascorbate reductase (Solyc09g009390.3.1), 2-Cys peroxiredoxin 1 (Solyc10g082030.2.1), Peroxiredoxin (Solyc07g042440.3.1), Late embryogenesis abundant protein (Solyc06g048840.3.1) and GDSL esterase (Solyc05g013690.3.1). Among them, Monodehydroascorbate reductase, Peroxiredoxin, and 2-Cys peroxiredoxin exhibited a decrease in abundance, while other proteins showed an increase in their abundance compared to control seedlings (Fig. 3 and Table 3).

Late embryogenesis protein has been described for responding to abiotic stress^78^, while GDSL esterase function in response to both biotic and abiotic stress^79^ 2-Cys peroxiredoxin 1 has been proven to protect the plant cells from oxidative damage^80^. In Arabidopsis, annexin group proteins act as a target for calcium signals and play a major role in stress response^81^. Monodehydroascorbate reductase is one of the key antioxidant enzymes involved in the scavenging of reactive oxygen species^82^. Peroxiredoxins (Prx) employ a thiol-based catalytic mechanism to reactive oxygen species and are major components of the antioxidant defense system in higher plants. Differential regulation of stress and defense-related proteins in the mutant indicated the involvement of PhyA in the stress-responsive process during tomato seedling’s early development, but the underlying mechanism needs to be further examined.

*Photorespiration* Light plays a significant role in the development of photorespiratory pathways^83^. However, the exact mechanism by which the phytochromes control photorespiration is not completely known. Photorespiratory pathways are meant for recycling the by-product of photosynthetic carbon assimilation, 2-phosphoglycolate back to ribulose 1,5-bisphosphate and it is an indispensable process in oxygenic photosynthesis^84,85^.

The proteomics data revealed a decreased abundance of proteins involved in photorespiration compared to their corresponding control seedlings. Glycolate oxidase, one of the key enzymes mediates photorespiratory metabolism is shown to be reduced its abundance in *fri* mutant (Fig. 2B and Table 3). Moreover, expression of both mitochondrial enzymes glycine decarboxylase complex and serine hydroxy methyltransferase^18,86^, which seems to be directly involved in photorespiration, showed a significant decrease in the accumulation in the mutant compared to the control seedlings, which is discussed in the previous section (Fig. 3 and Table 3). Our findings suggest that photorespiration in FR condition is regulated by PhyA and loss of PhyA activity may decrease the photorespiration rate.

*Other proteins and pathways* Proteins such as SOUL heme-binding family protein (Solyc07g061790.3.1) and leucine-rich repeat receptor-like protein kinase family (Solyc11g056680.1.1) that participate in various signaling pathways were shown to be deregulated in the mutant under FR treatment. In Arabidopsis, *AtHBP2* was identified as a phytochrome A-induced transcript that responds to light during de-etiolation^87^. *AtHBP2* encodes a SOUL protein and is proposed to have a role as a cytosolic tetrapyrrole-carrier protein^88^. Leucine-rich repeat receptor-like protein kinase family (LRR-RLK) are important mediators of cell–cell communication to transmit developmental cues and environmental stimuli and are also involved in defense/resistance response against pathogens^89–91^. The protein abundance of 2-oxoglutarate-dependent dioxygenase (2-ODD, Solyc01g006580.3.1) that is involved in the synthesis of plant hormones such as ethylene, GA, and flavonoids^92,93^ was found to be decreased compared to the control. Previous reports suggest that active phytochrome regulates the synthesis of gibberellin (GA), which promotes germination^94,95^. Differential regulation of 2-ODD shows the involvement of PhyA in regulating the hormone biosynthesis (Table 3).

### PhyA regulates protein abundance in the dark

To understand the specific role of PhyA during skotomorphogenesis, we profiled the proteome of dark-grown AC and *fri* seedling. A total of 36 statistically significant proteins were identified (Supplementary Fig. S5) from the iTRAQ-based quantitative temporal proteomic analysis of *fri* mutant grown under dark (*fri*/D versus AC/D). From these, 13 proteins were further selected based on the fold ratio values (≥ 1.5 fold up-regulation or ≤ 0.66 fold down-regulation) and biological relevance. Our results showed an alteration in the abundance number of proteins involved in photosynthesis, carbon assimilation, stress/ defense, and storage.

Proteins that are involved in photosynthesis and carbon assimilation such as PorB, Por isoform, PGK, Mg-protoporphyrin IX chelatase, and RuBP carboxylase small subunit exhibited a decrease in abundance in *fri* mutant when grown under dark compared to the control. Interestingly, PorB, PGK, and RuBP carboxylase small subunit proteins were found to be light-independent proteins (that we discussed previously in the manuscript) (Supplementary Fig. S5). Our results suggest the possibility of a PhyA action in the dark and that these proteins are under positive regulation of PhyA. We also found an increased accumulation of storage proteins such as Oil body-associated protein 1A and Legumin 11S-globulin in the mutant compared to the control when grown under dark. Storage proteins are generally expressed in the developing seeds and stored until the onset of photosynthesis^96^. The increased abundance of storage proteins in the mutant under dark indicates the slow breakdown of these proteins compared to control seedlings. Interestingly several proteins associated with stress/defense response such as LEA, Glutathione S-transferase, Major allergen Pru, and 2-Cys peroxiredoxin displayed a differential abundance in the mutant compared to AC seedling under dark.

As suggested by a number of mutant studies, the longer hypocotyl in *phyA* mutant in the dark when compared to their isogenic WT lines^4,37^, which indicates that phyA also functions in the dark. Previous reports propose that active phyA in the developing seed or in the fruit of the parent might induce downstream signaling and that would be active in germinating seed or seedling. Suppression of ABSCISIC ACID INSENSITIVE3 (ABI3) expression is normally mediated by seed-activated phyB and the absence of seed activated phyB in the mutant seedling resulted in an elevated level ABI3 in dark-grown Arabidopsis phyB seedlings^97^. In another word, light-activated phyA-mediated signaling occurred during seed development or in the parent plant is propagated to the germinating seed and seedling through an intermediary, such as ABI3. Although our study does not show the presence of an intermediate like ABI3, our results indicate that a PhyA is required for the expression of some of the light-independent proteins, and loss of PhyA resulted in decreased abundance of these proteins. Differential protein abundance in the *phy A* mutant compared to control seedlings grown in the dark strongly support the broad range of phyA’s function such as regulation of carbon flux, stress/defense, and conversion of storage proteins in dark-grown seedlings.

### PhyA mediated signaling regulates a large number of chloroplast associated proteins

One of the important goals of proteomics is to identify the functions of proteins in various cellular organelles and pathways. The identification of subcellular locations of proteins can furnish valuable insights for revealing their functions. Additionally, this information can be explored to understand how each protein interacts with others in cellular network systems. Overall, we used 57 proteins from our study, that are found to be statistically significant in various comparisons discussed previously in the manuscript, to further understand their subcellular localization pattern. The subcellular localization of these proteins was assessed using Plant-mPLoc. Our results showed that 45% of the proteins are predicted to be targeted to the chloroplast, 12.08% to the vacuole, 7.01% to mitochondria and nucleus, 5.26% to the cytoplasm, 3.5% to the cell wall, and 1.75% were targeted to the peroxisome. We also observed that 17.54% of the total proteins were present in more than one subcellular location (Table 1).

Most of the proteins involved in chlorophyll synthesis, photosynthesis, Calvin cycle, plastidial glycolysis, and some of the proteins that are involved in amino acid metabolism and fatty acid synthesis are targeted to the chloroplast. Storage proteins and proteases were assembled in the vacuole. In mitochondria, many of the proteins associated with nitrogen metabolism were accumulated, while in the nucleus, proteins associated with signaling were found. Defense/stress-related proteins were assembled in the cytoplasm (Table 1 and Fig. 5).

Upon absorption of red light, activated Phytochromes are translocated from the cytoplasm to the nucleus, where they inhibit the activity of several transcription factors, and thereby induce genome-wide changes in the transcription of target genes and mediate various light responses^98,99^. A recent study revealed that phytochrome controls not only the transcription but also the alternative splicing to mediate light responses in *A. thaliana*^100^. The genome-wide changes in alternative promoter selection modulate protein localization, thereby allowing plants to adapt to light environments. Proteins associated with chloroplast are the main targets of this regulatory mechanism, which is in line with the observations that phytochrome regulates most chloroplastic functions, including various aspects of photosynthesis^101,102^.

### Primary metabolite profiling using GC–MS reveals that PhyA regulates primary metabolism in tomato seedlings

**Figure 4:**
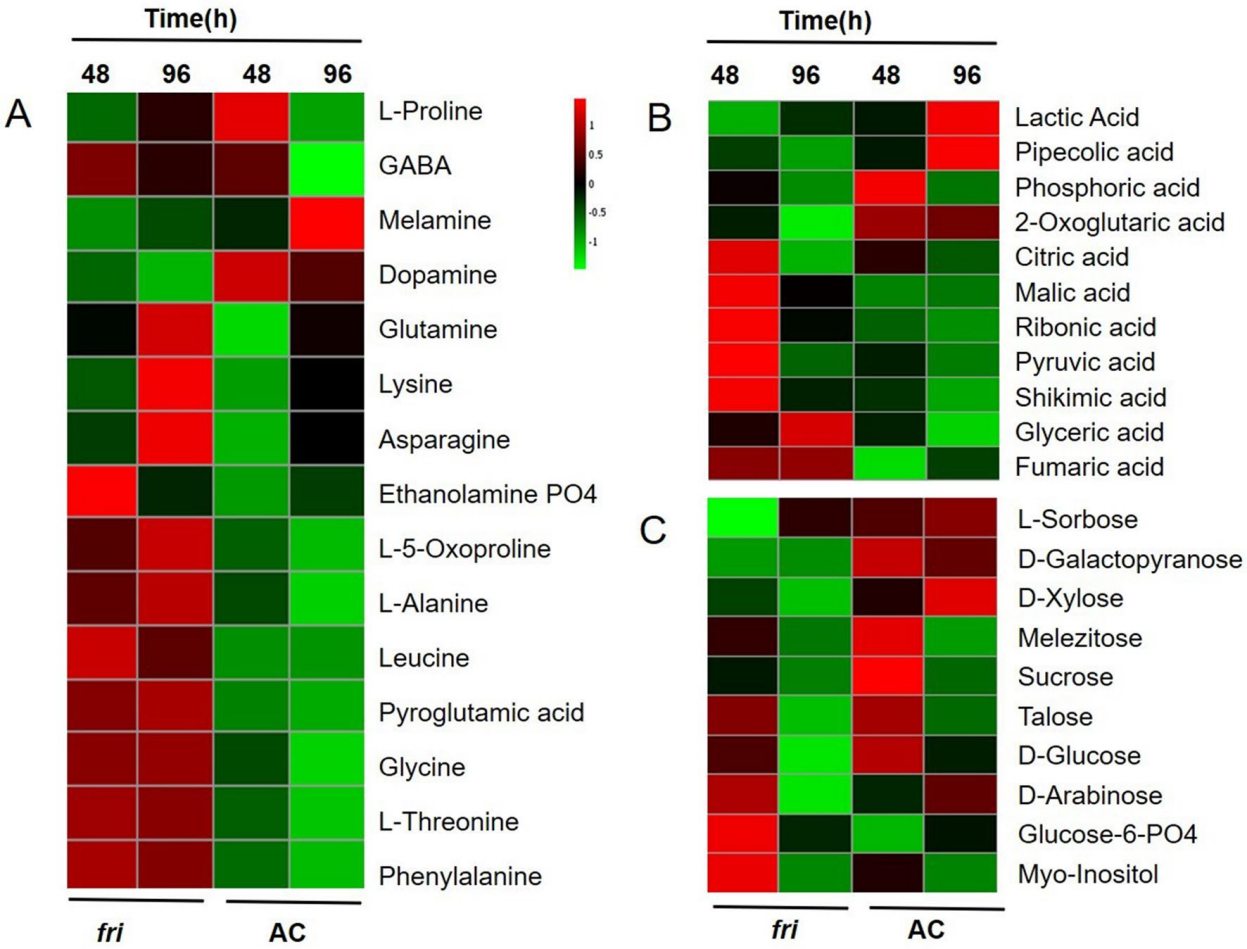
Metabolomic profiling of *fri* mutant to validate the findings from discovery proteomics. Heat map showing differential expression of (**A**) amino acids (**B**) organic acids (**C**) sugars in the seedlings treated under FR light compared to AC.

In the study, we used 4 replicates of AC and *fri* mutant seedlings of tomato, that are grown under FR light and dark in two-time points (48 and 96 h) (Fig. 1B) to understand the temporal variation in metabolite accumulation. A total of 77 compounds were identified from 4 replicates. Normalized abundance value of these metabolites at various treatment conditions, is shown in Supplementary Table S2. Statistical analysis was conducted in two groups; AC/FR versus AC/D and *fri*/FR versus AC/FR. 39 significant compounds were identified for AC/FR versus AC/D, and 49 compounds were identified for *fri*/FR versus AC/FR. The most prominent group of modulated metabolites belonged to organic sugars, followed by amino acids and organic acids (Fig. 4 and Supplementary Table S2). The mutation in PhyA resulted in a drastic variation in the profile of primary metabolites such as sugars, amino acids, and organic acids (Fig. 4 and Supplementary Fig. S6). Previous reports suggest that Phy signaling has substantial effects on the majority of primary carbon metabolic pathways and a subset of secondary metabolites. Metabolomics experiments comparing *Phy* mutants with wild-type controls in Arabidopsis and rice^7,8,11,50,56,103^ confirm the same. Even though the results differ, the general trend indicates that a large number of metabolites, particularly sugars and tricarboxylic acid cycle components, accumulate to higher levels in *phy* mutants compared to wild-type plants.

Jumtee et al.^7^ reported that FR exposure led to a PhyA dependent fall in the levels of sugars (including Glc, Fru, and Gral). As FR light activates de-etiolation but not greening, the carbon resources may be used for growth but not restored through photosynthesis under these conditions. However, studies conducted by a few other groups^8,56,104^ reported reduced sugar levels in *phy* mutants when compared to the wild type. These studies suggest that the impact of Phy signaling on sugar and starch abundance may be dependent on the developmental stage and/or experimental conditions. Patel et al.^103^ showed that the quality of light has a significant role in the accumulation of soluble sugars, especially sucrose. This is very clear from these findings that although Phy deficiency alters sugar levels, whether it leads to a rise or fall in sugars, will potentially depend on several factors.

We found an accumulation of several organic sugars in the mutant seedlings during 48 h of FR irradiation. Sugars such as arabinose, myoinositol, glucose 6-phosphate, and talose showed a significantly increased abundance. However, it declined drastically as the irradiation time increased from 48 to 96 h (Fig. 4C). The decreased accumulation is an important clue suggesting that the absence of PhyA-regulated biochemical pathways for several hours could lead to decreased carbon metabolism and the possibility of the sugars being used up for the survival of the mutant. Our metabolite profiling data showed an increased accumulation of several amino acids in *fri* mutant in the FR (Fig. 4A). We also observed that amino acid levels in the mutant showed no change in their levels over the FR irradiation period (48 to 96 h).

Additionally, we found a depletion in the amino acid pool in the AC seedling during irradiation (Supplementary Fig. S6). Our results corroborate the previous finding by Liu et al.^105^, where amino acid concentrations drop in wild-type but not in *phyA* mutant seedlings during FR-induced de-etiolation. This result indicates that this PhyA-mediated effect may arise from an increase in protein synthesis to support growth, which would deplete the amino acid pool. Increased accumulation of organic acids (Fig. 4B) was observed in the mutant under 48 h of FR irradiation; however, a drastic decline was observed under 96 h of treatment. Most of the organic sugars in AC seedlings under FR showed a decrease in their abundance. Previous transcript profiling studies revealed that enzymes catalyzing the synthesis of organic acids were down-regulated in *phyABDE* mutants^8^, suggesting that these metabolites are not elevated through transcriptional up-regulation. Another interpretation is that organic acids accumulate due to decreased synthetic processes that use these metabolites. Regarding the accumulation of organic acids and amino acids, it is currently unclear whether this is because of increased production or slower consumption^49^; however, our proteomics data showed a decreased abundance of several proteins associated with amino acid biosynthesis.

Our temporal proteomics analysis of phyA mutant revealed a wide-ranging influence of PHYA on a myriad of pathways during the onset of photomorphogenesis. PHYA seems to elicit metabolism change by regulating protein levels during heterotrophic to autotrophic transition. Of interest was a set of proteins exhibiting light-independent accumulation with a significant variation in their levels during the course of 96 h treatment. It remains to be determined whether the higher abundance of some of these proteins was due to the overall absence of turnover or specifically no turnover under FR.

**Figure 5:**
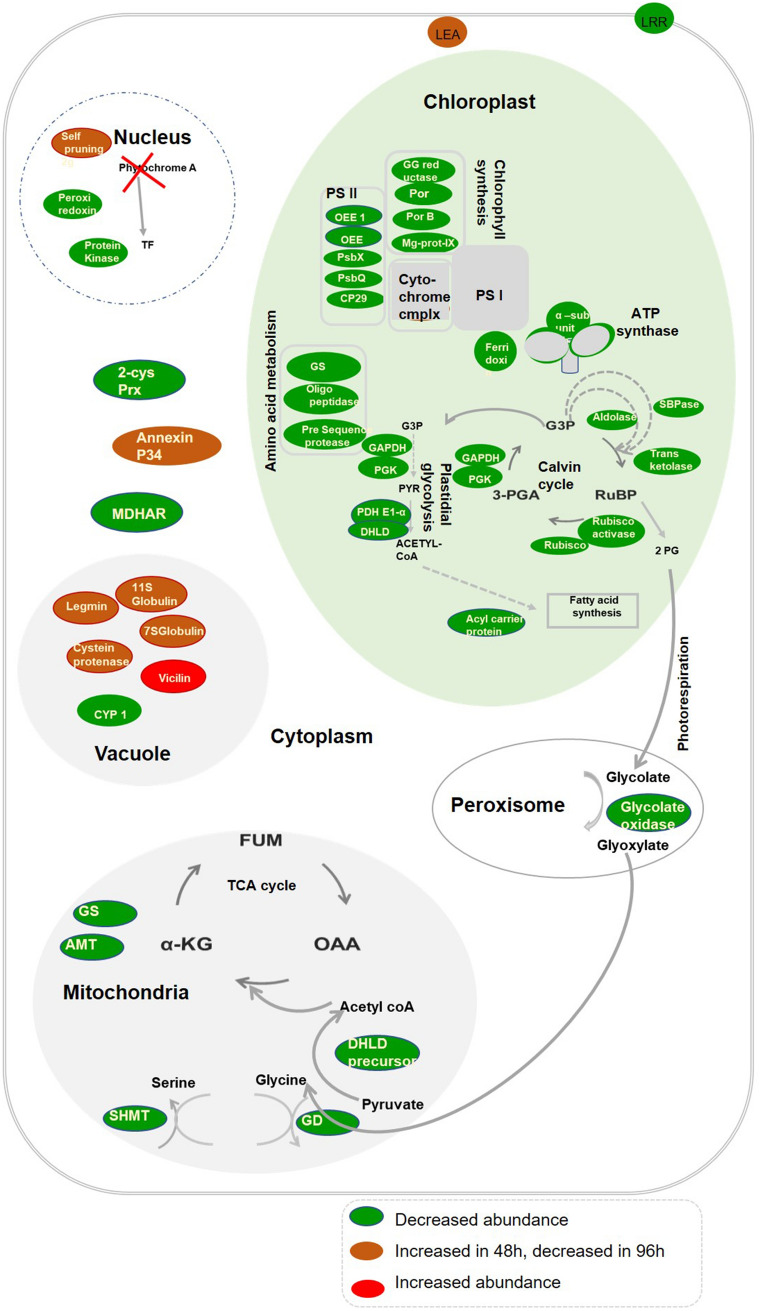
Schematic model of PhyA-regulated proteins in tomato seedling grown under FR light. The green ellipse represents proteins downregulated in *fri* mutant while red showed an upregulation. The brown ellipse represents proteins downregulated in 48 h but upregulated in 96 h.OEE1-33 kDa precursor protein of oxygen-evolving complex; OEE-The oxygen-evolving enhancer protein; DHLD precursor-dihydrolipoamide dehydrogenase precursor; PDH E1 α-Pyruvate dehydrogenase E1 component subunit alpha;α KG-*α*-Ketoglutaric acid; DHLD-dihydrolipoamide dehydrogenase; 2 PG-2-phosphoglycolate; Cyp 1-cysteine protease; Fum-fumaric acid; GAPDH-glyceraldehyde 3-phosphate dehydrogenase; PsbX-psbX photosystem II 23 kDa protein; PsbQ-photosystem II oxygen-evolving complex protein; SBPase-Sedoheptulose 1,7-bisphosphatase; G3P-glyceraldehyde-3 phosphate; PGK-phosphoglycerate kinase; 3-PGA-3-Phosphoglyceric acid; Pyr-pyruvate; RuBP-RuBP: ribulose-1,5bisphosphate; 2-Cys Prx-2-cysteine peroxiredoxin; Mg-prot-IX-Mg-protoporphyrin IX chelatase; GG reductase-Geranylgeranyl reductase; PorB-light-dependent NADH:protochlorophyllide oxidoreductase; Por-light-dependent NADH:protochlorophyllide oxidoreductase 1; MDHAR-Monodehydroascorbate reductase; Aldolases-(Fructose-bisphosphate aldolase1, Fructose-bisphosphate aldolase2), α sub unit-ATP synthase subunit alpha, GS-chloroplast glutamine synthetase; AMT-Amino methyltransferase; SHMT-Serine hydroxymethyltransferase; GD-glycine decarboxylase; OAA-Oxaloacetate.

In conclusion, an integrated protein and metabolite investigation revealed that loss of PHYA significantly impacts on photosynthesis, carbohydrate metabolism, photorespiration, and other metabolic pathways under FR irradiation. The proteomic profiling of tomato phyA mutant delineate its role during FR-mediated photomorphogenesis. The reduced abundance of proteins in the mutant suggests an important role of phyA during FR-mediated growth and development, where it likely interacts with other signaling pathways to regulate the final response (Fig. 5). Overall, our study reveals that phyA through its network of interacting proteins plays a major role in the FR mediated photomorphogenesis.

## Methods

### Plant materials and growth condition

The seeds of tomato *cv*. Ailsa Craig (AC) and *fri* mutant lines carrying a mutation in *PHY-A*^37^ were used for this study. The tomato seeds used in this study are publicly available from TGRC, Davis, California. We have complied with all legislation for use of this material. Tomato seeds were surface sterilized in 2% (v/v) sodium hypochlorite for 15–20 min, followed by thoroughly washing under running tap water. The surface-sterilized seeds were then transferred on wet germination paper and kept in the dark at 25 ± 1 °C for seed germination. The germinated seeds were transferred to germination boxes lined with wet germination paper, were either kept in dark or under continuous FR (3 μmol/m^2^/s) at 25 ± 1 °C in a culture room. Later, the shoot tissue was harvested after 48 h and 96 h. After harvest, seedling tissue was immediately snap-frozen using liquid nitrogen and stored at − 80 °C until use. All of the above experiments were carried out in the darkroom and safe green light was used for the selection of germinated seeds and their transfer on germination paper (sterilized and soaked in autoclaved double distilled water).

### Protein extraction and digestion

Total protein was extracted from tomato AC and *fri* mutant seedlings grown for 48 and 96 h under FR light and dark using Urea extraction protocol^106^ with slight modification. The seedling samples (∼100 mg each) were ground to a fine powder using a pestle and mortar in liquid nitrogen, and the frozen powder was then extracted with a urea-based lysis buffer containing 8 M Urea, 50 mM Tris pH 8.0, 75 mM NaCl, and 1 mM MgCl_2_. Tissue lysis was performed with sonication at 40% amplitude, 5 s pulse on, and 5 s pulse of 2 min and 30 s (big probe-Sonics Vibra Cell) by placing the tube on ice throughout the procedure. After the sonication, bead milling (Bertin, MiniLys) at 90 s for 3 cycles was performed by adding 100 mg of beads (1.0 mm dia. ZIRCONIA/SILICA, BioSpec products, Cat No. 11079110z) to the tissue lysate in 1.5 ml/0.5 ml Eppendorf tubes. Tubes were then centrifuged to get rid of the debris at 8000 rpm (small rotor, Thermo centrifuge) for 15 min at 4 °C. Clear supernatant (200–250 µl) was collected in a fresh tube. Protein estimation was done using a 2D Quant kit (BioRad) as per the manufacturer’s protocol. The amount of protein considered for further downstream processing is 100 μg unless stated otherwise.

### iTRAQ labeling and fractionation

100 μg protein from each sample was digested into peptides with trypsin (Pierce, Madison, USA) for 16 h at 37 °C, followed by vacuum drying the peptides. Peptides were subsequently cleaned up with C18 desalting columns (Sigma, USA), and then iTRAQ labelling^107^ was performed according to the manufacturer’s instructions for the iTRAQ reagents 4-plex kit (AB Sciex Inc., Foster City, California, USA). AC 48 and AC 96 were labeled with iTRAQ tags 114 and 115 respectively, and *fri*48 and 96 were labeled with iTRAQ tags 116 and 117 (Supplementary Fig. S1). For each sample, three independent biological replicates were performed. After labeling, samples from the same set were combined and lyophilized. The peptide mixtures were dissolved in 0.1% TFA. The samples were fractionated using a gradient of ACN and triethylamine by eluting the peptide with a linear gradient of 10–90% ACN in triethylamine. 7 fractions were collected and lyophilized prior to the LC − MS/MS analysis. The peptides were quantified using Thermo plate reader and 1ug of peptide was used for the LC–MS/MS run.

### LC–MS/MS analysis

Each fraction was resuspended in 0.1% formic acid. MS/MS analysis was performed on a hybrid quadrupole Orbitrap (Q Exactive) mass spectrometer (Thermo Fisher Scientific, Bremen, Germany) with high energy collision dissociation (HCD). The MS system was equipped with an automated Easy-nLC 1000 system (Thermo Fisher Scientific, Germerling, Germany). A linear gradient of solvent A (0.1% formic acid) to solvent B (0.1% formic acid, 99.9% acetonitrile) was run for 95 min, followed by a ramp to 98% B for 5 min. The instrument was run in a positive mode applying data-dependent MS scan and MS/MS acquisition^108^. Full scan MS spectra (400–2000 m/z) were acquired with resolution R = 70,000, 170,000 intensity thresholds, and fixed first mass = 105 m/z.

### Protein identification and data analysis

Proteome Discoverer 1.4 with the SEQUEST algorithm (Thermo Scientific Inc., Bremen, Germany) was used to search the original MS/MS protein data in the specified non-redundant databases. Protein identification was executed against the SGN^109^ tomato database ITAG3.0. Static modification for the iTRAQ 4-plex run was set to Carbamidomethyl and Oxidation of Methionine as a dynamic modification. Proteome Discoverer nodes for spectrum grouper and spectrum selector were set using default parameters. Tolerances were set to a 10 ppm precursor mass tolerance and a 0.05 Da fragment mass tolerance. 1 maximum missed cleavage sites of trypsin digestion was allowed. Percolator was used for protein identification with parameters set with a strict target false discovery rate (FDR) at 0.01 and a relaxed target FDR at 0.05. The mass spectrometry proteomics data have been deposited to the ProteomeXchange Consortium via the PRIDE^110^ partner repository with the dataset identifier PXD023589.

### Protein networks and functional analysis

Pathway mapping of the differentially regulated proteins (*p* < 0.05) was conducted using the KEGG PATHWAY tool available from the Kyoto Encyclopedia of Genes and Genomes (https://www.genome.jp/kegg/tool/map_pathway2.html)^29^. Metaboanalyst (https://www.metaboanalyst.ca/)^28^ was used for the generation of heat-map.

### Metabolite profiling

For GC–MS based metabolite analysis, we used the same tissue samples from AC and *fri*, which were previously used for our proteomics study. The metabolites were extracted, analyzed, and identified using the protocol previously described in the^111^. In brief, the polar metabolite fraction was extracted from 100 mg of dry tissue powder by adding mixed with 1.4 ml 100% methanol and 60 μl of internal standard ribitol (0.2 mg/ml, w/v) and shaken at 70 °C in a thermomixer for 15 min at 650 rpm. The extract was then mixed with an equal amount of MilliQ water, centrifuged at 2200*g* at 25 °C, and the supernatant was collected. The 150 μl of supernatant was dried in the vacuum for 2 h. The sample was dissolved in 80 μl of pyridine containing 20 mg/ml methoxyamine hydrochloride and kept at 37 °C for 90 min at 650 rpm. Then the samples were derivatized by adding 80 μl of *N*-trimethylsilyl-*N*-methyl trifluoroacetamide (MSTFA) and the mixture was incubated at 37 °C for 30 min at 650 rpm.

The derivatized samples were analyzed using LECO-PEGASUS GCXGC-TOF–MS system (LECO Corporation, USA) equipped with 30 m Rxi-5 ms column with 0.25 mm internal diameter and 0.25 μm film thickness (Restek, USA). The injection temperature, interface, and ion source were set at 230, 250, and 200 °C respectively. For separation of groups of metabolites, the following program was used; isothermal heating at 70 °C for 5 min, followed by 5 °C/min oven temperature ramp to 290 °C, and final heating at 290 °C for 5 min. A 1 μl of the sample was injected in splitless mode, and mass spectra were recorded at 2 scans/s within a mass-range from 70 to 600.

### Identification of metabolites

The NetCDF files obtained from ChromaTOF software 4.50.8.0 chromatography version (LECO Corporation, USA) were analyzed using MetAlign 3.0 (www.metalign.nl) software^112^ with a signal to noise ratio of ≥ 2, for baseline correction, noise estimation, alignment, and extraction of ion wise mass signal. The MetAlign results were further processed with MS Clust^113^ software for the reduction of data and compound mass extraction. NIST MS Search v 2.2 software was used for the identification of compounds with the NIST (National Institute of Standard and Technology) Library and Golm Metabolome Database Library (http://gmd.mpimp-golm.mpg.de/)^114^. The compound hits with maximum matching factor (MF) value (> 700), and the least deviation from the retention index (RI) was used for metabolite identity. The metabolite levels were quantified by normalizing with the concentration of the internal standard (ribitol).

### Statistical analysis

In all experiments, 3 biological replicates were used, and the mean with the standard error was calculated. For statistical analysis of metabolite data, including PCA, we used online software MetaboAnalyst 4.0 (http://www.metaboanalyst.ca/)^28^.

## Supplementary Information

Supplementary Information 1.

Supplementary Information 2.

Supplementary Information 3.
