# Supplementary material for: iTRAQ-based proteome profiling revealed the role of Phytochrome A in regulating primary metabolism in tomato seedling
Source: Sci Rep. 2021 Apr 6;11:7540. doi: 10.1038/s41598-021-87208-9 (PMC8024257; doi:10.1038/s41598-021-87208-9)
Supplement: Supplementary file 1 — Supplementary Information 1. [file 41598_2021_87208_MOESM1_ESM.docx]

**Original Research Article**

**iTRAQ-based proteome profiling revealed the role of Phytochrome A in regulating primary metabolism in tomato seedling**

Sherinmol Thomas^1^, Rakesh Kumar^2,3^, Kapil Sharma^2^, Abhilash Barpanda^1^, Yellamaraju Sreelakshmi^2^, Rameshwar Sharma ^2^, and Sanjeeva Srivastava^1*^

^1^Proteomics Lab, Department of Biosciences & Bioengineering, IIT Bombay, Mumbai, 400076, Maharashtra, India.

^2^Repository of Tomato Genomics Resources, Department of Plant Sciences, University of Hyderabad, Hyderabad, 500046 India.

^3^Deptartment of Life Science, Central University of Karnataka, Kadaganchi, Kalaburagi, 585367, Karnataka, India.

^*^Correspondence: Prof. Sanjeeva Srivastava, E-mail: [sanjeeva@iitb.ac.in](mailto:sanjeeva@iitb.ac.in)

Phone: +91-22-2576-7779, Fax: +91-22-2572-3480

| **Title** | **Content** |
| --- | --- |
| **Supplementary Figure S1** | **Details of iTRAQ labelling strategy** |
| **Supplementary Figure S2** | **A: Gene ontology analysis; classification according to molecular function**  **B: Gene ontology analysis; classification according to cellular components** |
| **Supplementary Figure S3** | **Statistical analysis_AC during FR treatment in comparison to dark**  **A: One-way Anova**  **B: PLSDA- plot** |
| **Supplementary Figure S4** | **Statistical analysis_*fri* mutant during FR treatment in comparison to AC**  **A: One-way Anova**  **B: PLSDA- plot** |
| **Supplementary Figure S5** | **PhyA regulated protein expression during growth under dark** |
| **Supplementary Figure S6** | **Heat map showing altered metabolite profile in response to FR irradiation in AC seedlings** |


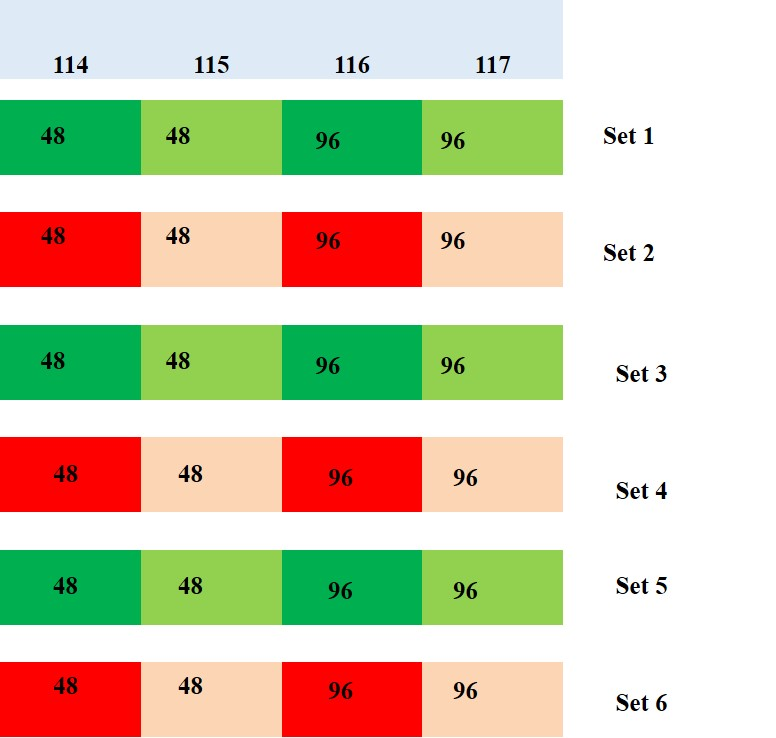

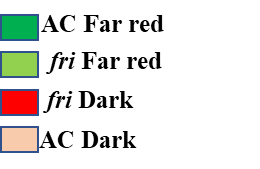


**Supplementary Figure S1:** Details of iTRAQ labelling strategy.

a b c

a b c

A

B


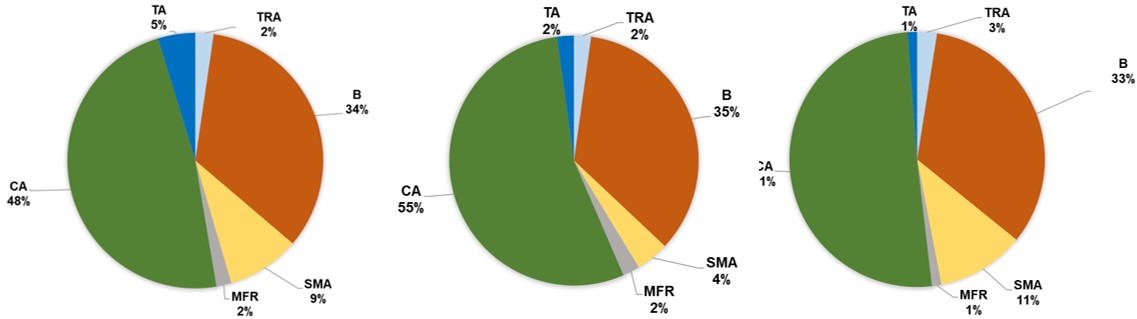


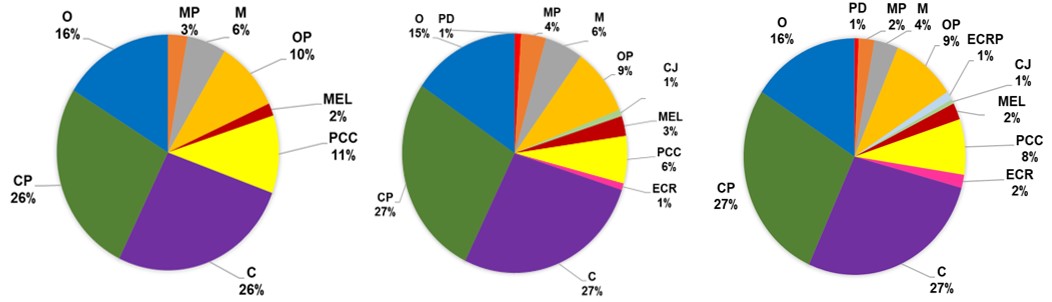


**Supplementary Figure S2: (A) Gene ontology analysis; classification according to molecular function.** (a)in all the identified proteins(b) significant proteins identified in the comparison of Dark vs FR in Ailsa craig(c) significant proteins identified in the comparison of Ailsa craig vs *fri* mutant grown under FR light. TRA (translation regulator activity), B(binding), SMA (structural molecule activity), MFR (molecular function regulator), CA (catalytic activity), TA (transporter activity). **(B) Gene ontology analysis; classification according to cellular components.** (a)in all the identified proteins(b)significant proteins identified in the comparison of Dark vs FR in Ailsa craig(c)significant proteins identified in the comparison of Ailsa craig vs *fri* mutant grown under FR light. MP (membrane part), M(membrane), OP (organelle part), MEL (membrane-enclosed lumen), PCC (protein-containing complex), C(cell), CP (cell part), O(organelle), PD (plasmodesma), ECR (extracellular region), CJ (cell junction), ECRP (extracellular region part)


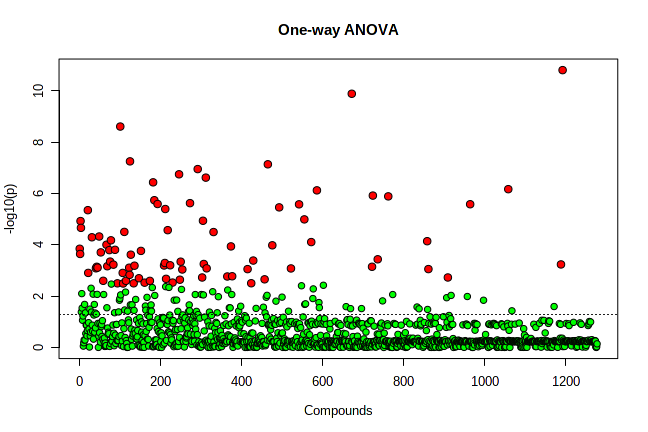


A B


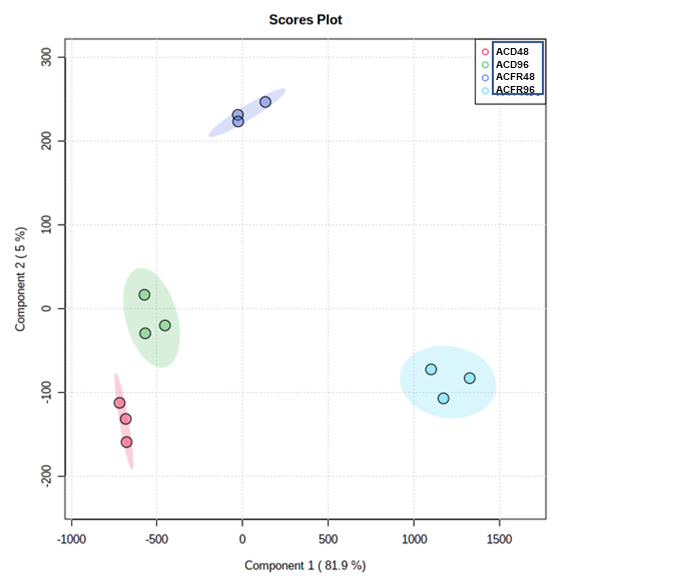


**Supplementary Figure S3:** One-way Anova shows 85 statically significant proteins in an analysis of FR grown AC seedlings in comparison to dark at two time points (A). PLS-DA plot is showing segregation among the different time points of FR treatment of *fri* mutant and AC groups based on the protein fold ratio values in each group. Based on the differences among the protein fold ratio values on a 2D-score plot, the four groups that are segregated in AC/FR versus AC/D group analysis were ACD48, ACD96, ACFR48, and ACFR96 The three points in each data group represent the three independent biological replicates (B).


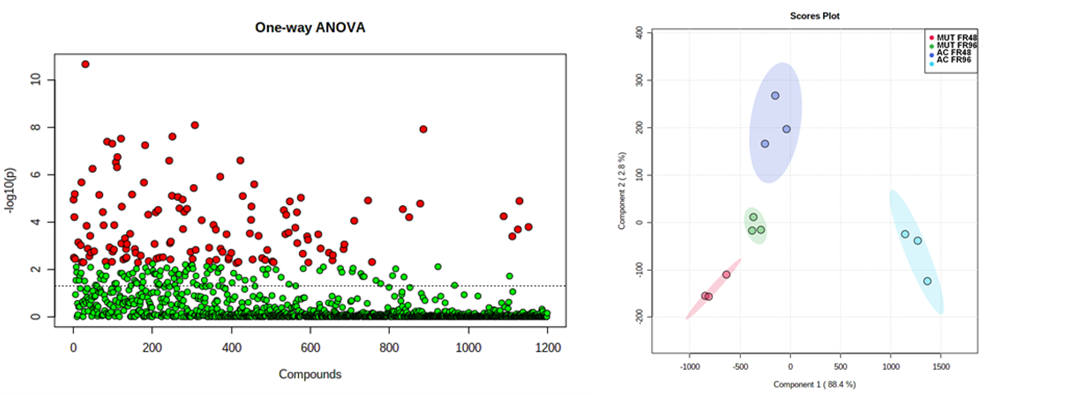


A B

**Supplementary Figure S4:** One-way Anova shows 135 statically significant proteins in an analysis of *fri* mutant in comparison to AC at two time points grown under FR light (A). PLS-DA plot is showing segregation among 4 different groups of the different time point treatment of FR grown *fri* mutant in comparison to AC seedlings based on the protein fold ratio values in each group. Based on the differences among the protein fold ratio values on a 2D-score plot, the four groups that are segregated in *fri*/FR versus AC/FR group analysis were MUTFR48, MUTFR 96, ACFR48, and ACFR96.The three points in each data group represent the three independent biological replicates(B).


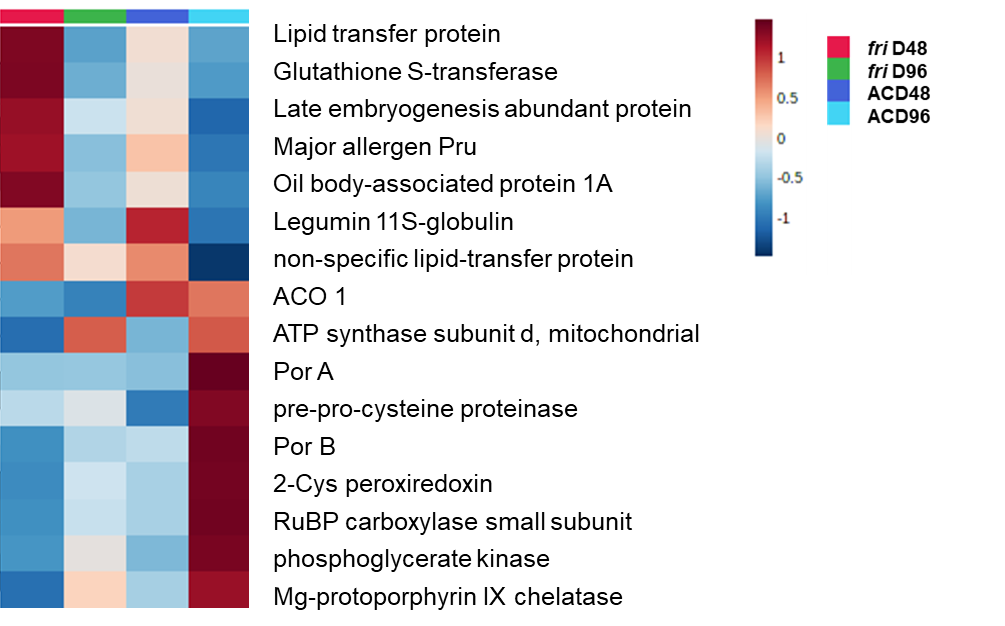

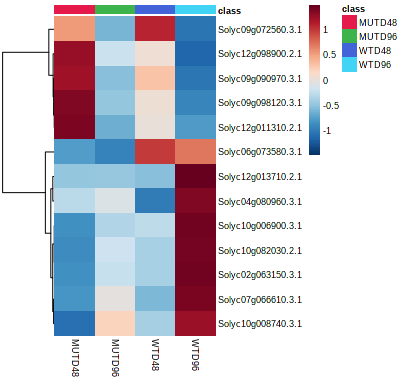


Legumin 11S-globulin

Late embryogenesis abundant protein

Major allergen Pru

Oil body-associated protein 1A

Glutathione S-transferase

ACO 1

Por B

pre-pro-cysteine proteinase

Por

2-Cys peroxiredoxin

RuBP carboxylase small subunit

phosphoglycerate kinase

Mg-protoporphyrin IX chelatase

**Supplementary Figure S5. PhyA regulated protein expression during growth under dark.** Heat map showing the fold ratio of differentially altered proteins involved in nitrogen metabolism, storage, photosynthesis, carbon metabolism and stress/ defense metabolic pathways in *fri* mutant in comparison to AC seedlings under dark. Por B- light-dependent NADH: protochlorophyllide oxidoreductase; Por- light-dependent NADH: protochlorophyllide oxidoreductase 1

**
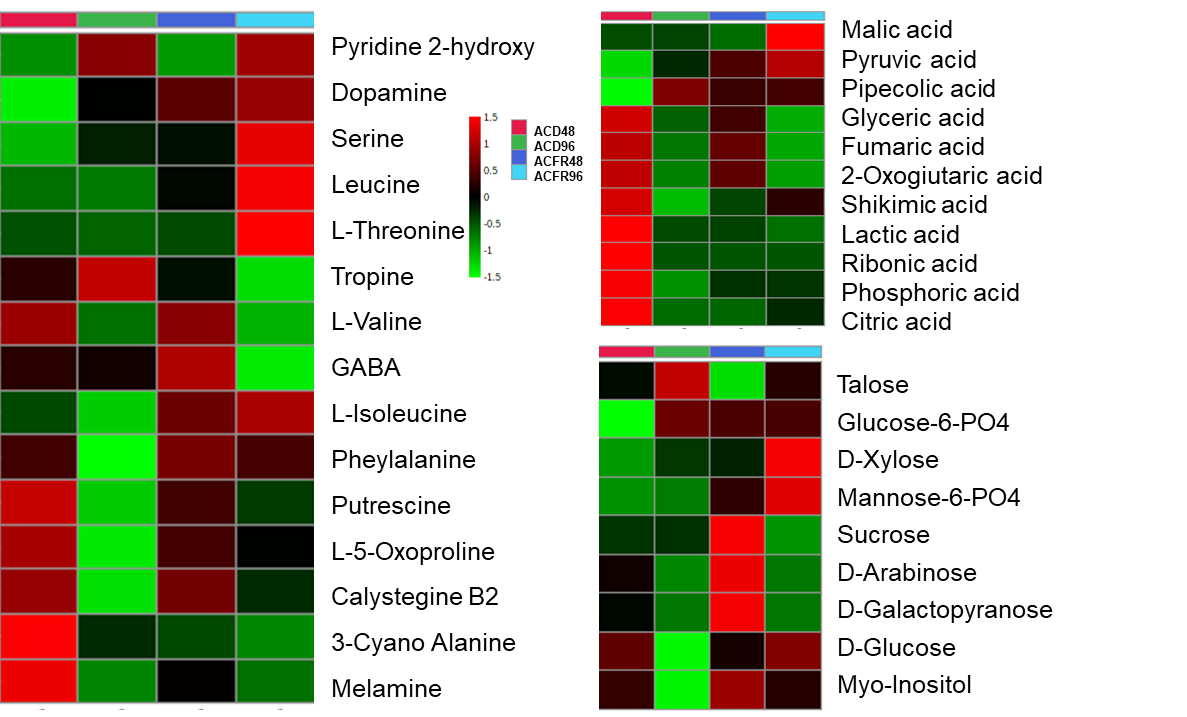
**

**Supplementary Figure S6:** Heat map showing the differentially altered metabolites in response to FR irradiation in AC seedlings.

**Supplementary Information:**

**Supplementary Table S1:** Raw abundance, normalized abundance, grouped abundance and abundance ratio of total identified proteins at various treatment conditions, is shown in Supplementary Table S1

**Supplementary Table S2:** Normalized abundance value of identified metabolites at various treatment conditions, is shown in Supplementary Table S2.
